# A transcription regulator atlas identifies TOX3 as an Atoh1 coactivator in cerebellar development and tumorigenesis

**DOI:** 10.1073/pnas.2527163123

**Published:** 2026-03-18

**Authors:** Xiaoxin Chen, Xiaochen Zhong, William Yue, Bruce Wang, Brian Woo, Hani Goodarzi, Zaili Luo, Q. Richard Lu, Frédéric Flamant, Jeremy F. Reiter, Guo N. Huang

**Affiliations:** ^a^Cardiovascular Research Institute, University of California San Francisco, San Francisco, CA 94158; ^b^Department of Physiology, University of California San Francisco, San Francisco, CA 94158; ^c^Eli and Edythe Broad Center for Regeneration Medicine and Stem Cell Research, University of California San Francisco, San Francisco, CA 94158; ^d^Department of Medicine and Division of Gastroenterology, University of California San Francisco, San Francisco, CA 94143; ^e^Department of Biochemistry and Biophysics, University of California San Francisco, San Francisco, CA 94158; ^f^Department of Pediatrics, University of Alabama at Birmingham, Birmingham, AL 35294; ^g^Brain Tumor Center, Division of Experimental Hematology and Cancer Biology, Cincinnati Children’s Hospital Medical Center, Cincinnati, OH 45229; ^h^Ecole Normale Supérieure de Lyon, Institut National de Recherche pour l’Agriculture, l’Alimentation et l’Environnement (INRAE), CNRS, Institut de Génomique Fonctionnelle de Lyon, Lyon 69007, France; ^i^Chan Zuckerberg Biohub, San Francisco, CA 94158

**Keywords:** cerebellar development, medulloblastoma, granule neuron progenitors, transcription factor, TOX3

## Abstract

Organ development and function are orchestrated by intricate transcriptional circuits. Here, we present a comprehensive atlas profiling 1,904 transcription regulators in the brain, cerebellum, heart, kidney, liver, ovary, and testis of fetal, neonatal, and adult mice. Using this dataset, we uncover Thymocyte Selection-Associated High Mobility Group Box Family Member 3 (TOX3) as a potential coactivator of Atoh1 in cerebellar granule neuron progenitors (GNPs). *Tox3*-deficient mice display severe ataxia and cerebellar hypoplasia, driven by depletion of GNPs, diminished *Atoh1* expression, and impaired primary cilia. Single-nucleus RNA-sequencing analyses reveals compromised maintenance of the progenitor pool. *TOX3* is also highly expressed in subsets of medulloblastoma, and its deletion reduces cerebellar neoplasia and prolongs survival in a mouse model. Mechanistically, how lineage-defining factors such as Atoh1 drive robust gene expression despite weak intrinsic transactivation activity remains unclear. We show that Tox3 physically associates with Atoh1 and co-occupies shared regulatory elements, converting an otherwise weak single-copy Atoh1-responsive E-box into a highly active enhancer that drives transcriptional activation by up to 120-fold, including at an ultraconserved E-box downstream of *Atoh1* itself. Cross-species single-cell comparisons further show an association between *Tox3* expression and cerebellum expansion during vertebrate evolution. Together, this work supports Tox3 as a critical Atoh1 coactivator in cerebellar development, tumorigenesis, and evolution, while providing an atlas and screening strategy as a valuable resource for exploring novel transcriptional regulators in organogenesis and tissue physiology.

## Significance

The formation and function of organs depend on tightly controlled gene networks, yet we lack a comprehensive understanding of the transcription factors that drive organ development and disease. Here, we profile nearly 2,000 transcriptional regulators across multiple mouse organs and identify Tox3 as a key coactivator of Atoh1 in the cerebellum. Loss of *Tox3* disrupts cerebellar development, impairs progenitor maintenance, and reduces tumor formation in a mouse model of medulloblastoma. Mechanistically, Tox3 and Atoh1 synergistically activate target genes, revealing how weak transcription factors achieve robust gene expression. These findings uncover a fundamental mechanism of neuronal development and tumorigenesis and provide a resource and strategy for discovering novel transcriptional regulators in organogenesis and tissue function.

The formation and proper functioning of organs depend on complex transcriptional networks, yet how these networks achieve both robustness and context specificity in vivo remains incompletely understood. Organogenesis is driven by progenitor populations that must precisely balance proliferation with differentiation, a process coordinated by lineage-defining transcription factors acting together with cofactors that modulate their activity. While many lineage factors are essential for cell fate specification, they often possess limited intrinsic transactivation capacity, raising a fundamental question: how are stable, high-output transcriptional programs generated from inherently weak regulatory inputs?

This question is particularly salient during transient phases of progenitor amplification, when modest transcriptional activity must sustain rapid tissue growth. In the developing nervous system, such amplification underlies dramatic postnatal expansion and contributes to species-specific differences in organ size and complexity. The cerebellum exemplifies this challenge. Containing more than half of the neurons in the human brain, the cerebellum undergoes rapid postnatal growth driven largely by granule neuron progenitors (GNPs). These progenitors arise from the embryonic rhombic lip, expand extensively within the external granule layer, and subsequently differentiate and migrate inward to form the internal granular layer (1–4). This amplification phase is prominent in mammals and birds but reduced or absent in aquatic vertebrates, and variation in its duration and magnitude has been implicated in shaping cerebellar size, foliation, and evolutionary complexity (5, 6). Disruption of GNP proliferation or maintenance leads to cerebellar hypoplasia and ataxia (7), whereas uncontrolled expansion gives rise to medulloblastoma, the most common malignant pediatric brain tumor (8–10).

The basic helix–loop–helix (bHLH) transcription factor Atoh1 (also known as Math1) is indispensable for GNP specification, and its loss results in cerebella devoid of GNs, foliation, or lamination (11). Atoh1 also plays an essential role in GNP proliferation during both cerebellar development and in certain types of medulloblastoma, in part by controlling cilia formation and Sonic hedgehog (Shh) signaling (12). Despite its central role, Atoh1 exhibits weak transactivation activity as it induces only a modest (~twofold) activation of reporter gene expression driven by a single copy of its E-box binding motif (13). Thus, the potency of the Atoh1-dependent developmental program cannot be explained solely by its intrinsic biochemical activity, suggesting that additional regulatory mechanisms must stabilize and amplify Atoh1-driven transcription in vivo.

To systematically identify such mechanisms, we built a transcription regulator atlas by profiling 1,904 transcription factors and cofactors across developing mammalian organs. This comparative, organ-wise approach was designed to uncover regulators that confer context-dependent logic to conserved lineage programs. From this unbiased analysis, we identified TOX3, a high-mobility group (HMG) domain-containing protein, as a top cerebellum-enriched candidate. Although TOX3 polymorphisms have been linked to breast cancer and neurological disorders such as Parkinson’s disease and restless legs syndrome (14–18), its function in cerebellar development remains unknown.

Here, we identify TOX3 as a context-specific regulator of the cerebellar transcriptional program. Rather than functioning as a generic chromatin factor, TOX3 interacts with Atoh1 to coactivate shared target genes, including an ultraconserved enhancer downstream of the *Atoh1* locus, establishing a positive-feedback loop that sustains *Atoh1* expression and stabilizes GNP identity during rapid cerebellar expansion. Using genetic models, bulk and single-nucleus RNA-seq, ChIP-seq, reporter assays, and tumor models, we demonstrate that disruption of this network impairs progenitor maintenance, cerebellar growth, and Shh-driven tumorigenesis. Together, these findings offer insight into cerebellar development, disease, and the evolutionary emergence of progenitor amplification in vertebrates.

## Results

### Identification and Expression of an Understudied Transcriptional Regulator Tox3 in Cerebellar Development.

**Fig. 1.**
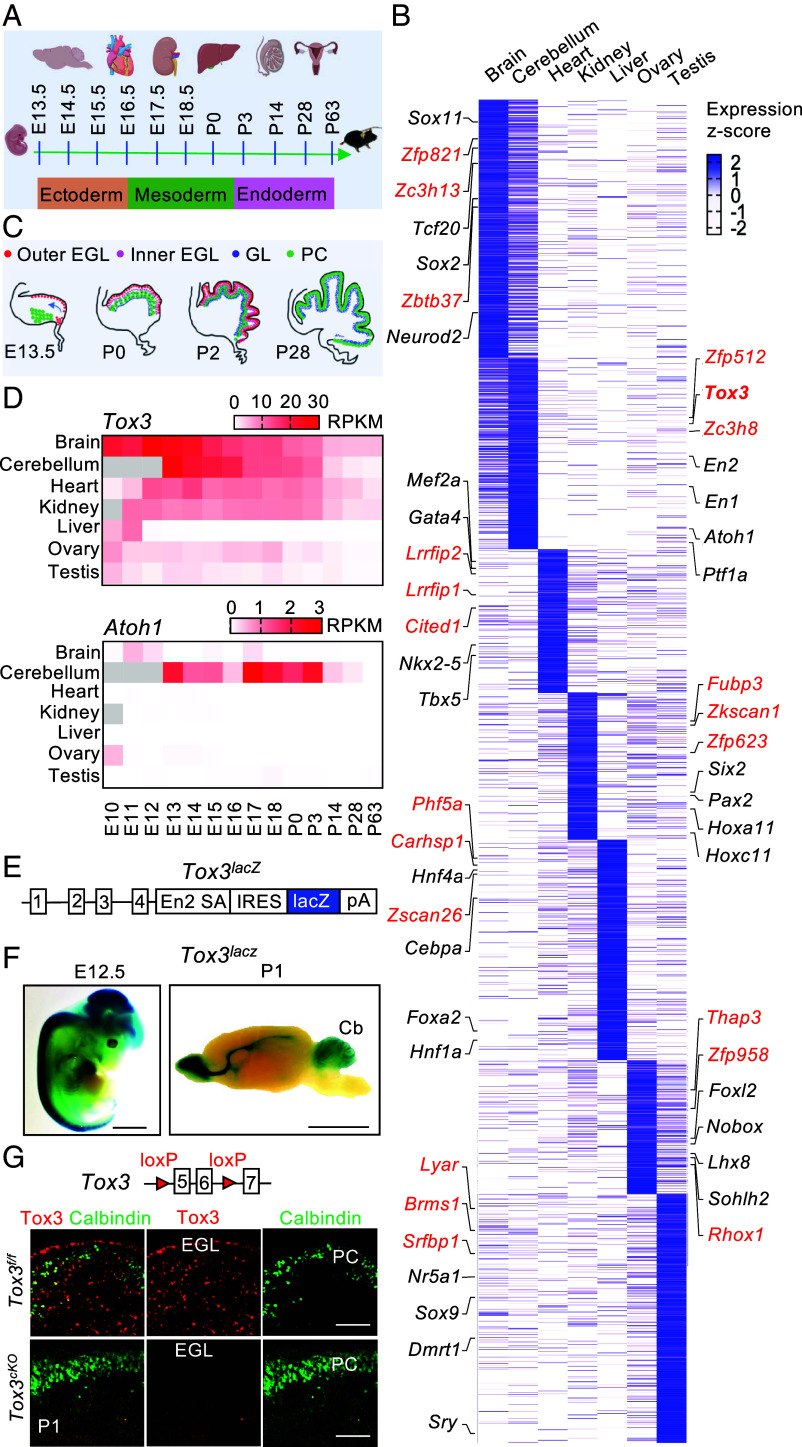
Identification and expression of an understudied transcriptional regulator, Tox3, during cerebellar development. (*A*) Mouse organs and developmental stages sampled for bulk RNA sequencing. E, embryonic; P, postnatal. (*B*) Heatmap showing organ-enriched transcriptional regulators grouped by organ and arranged by average gene expression levels across 14 developmental stages. Genes whose knockout phenotypes have not been reported are shown in red, whereas those with characterized knockout phenotypes are shown in black. (*C*) Schematic diagram illustrating stages of mouse cerebellar development, highlighting GNP proliferation and migration between E13.5 and P28. (*D*) Heatmap showing the expression of *Tox3 and Atoh1* in major mouse organs across developmental stages. Gray indicates unavailable data. (*E*) Schematic showing the mouse *Tox3* locus with insertion of the *lacZ* reporter gene. (*F*) X-gal staining of an E12.5 mouse embryo and P1 mouse brain. (*G*) Representative images of Tox3 and calbindin immunostaining in P1 control (n = 2) and mutant (n = 3) cerebella. EGL, external granule layer; GL, granule layer; PC, Purkinje cells. *Tox3^cKO^*, *Nestin-Cre;Tox3^f/f^*. [Scale bar: 2 mm (*F*), 100 μm (*G*).]

Transcriptional networks that drive organogenesis must maintain stable gene expression programs despite rapid cell proliferation and dynamic developmental cues. To identify regulators that may contribute to this transcriptional robustness, we performed an unbiased, organ-wise comparative transcriptomic analysis of transcription factors and cofactors across developing mouse tissues using a published RNA-seq dataset (19) (Fig. 1*A*). Through this analysis, we scrutinized the expression profiles of 1,906 annotated mouse transcription factors and cofactors (20), identifying 366, 272, 204, 208, 313, 190, and 353 genes enriched in the brain, cerebellum, heart, kidney, liver, ovary, and testis, respectively (Fig. 1*B* and Dataset S1). Gene Ontology (GO) analysis of these tissue-enriched transcriptional regulators unveiled enrichment in pathways closely associated with the developmental processes and biological functions of their respective organs (*SI Appendix*, Fig. S1*A*). Our analysis identified numerous genes with well-established roles in organ development (Fig. 1*B*). The complete list of characterized and uncharacterized genes is provided in Dataset S1. Collectively, these findings provide a comprehensive view of transcriptional regulators underlying organ development.

Considering that GNP amplification occurs predominantly during early postnatal stages (Fig. 1*C*), we further screened the 272 cerebellum-enriched genes to identify candidates with potential roles in this process. Using stringent criteria: an average Reads Per Kilobase per Million mapped reads (RPKM) > 1 and a postnatal day 0 (P0)/P63 expression ratio > 8, we identified 26 candidate genes (*SI Appendix*, Fig. S1*B* and Dataset S2). Because lampreys represent the earliest vertebrates to possess a primitive cerebellum (21), we hypothesized that genes contributing to cerebellar expansion arose after the divergence from lampreys and therefore lack clear lamprey orthologs. Consistent with this hypothesis, among the 26 candidates, 12 were classified as Type I genes, which have evolutionarily related but nonorthologous counterparts in lampreys, whereas the remaining 10 were classified as Type II genes, for which no similar lamprey genes were identified. Notably, *Tox3* emerged as a prominent Type I gene, exhibiting high expression during the perinatal period (RPKM >10).

Further investigation into the expression pattern of *Tox3* across seven organs in mice and humans using a published RNA-seq dataset (19) revealed intriguing insights. In mice, *Tox3* expression increased in the cerebellum between embryonic day 13.5 (E13.5) and E16.5, with a decline observed by P14 (Fig. 1*D*). Similarly, in humans, *TOX3* shows robust expression in the cerebellum from as early as 4 wk postconception through infancy (*SI Appendix*, Fig. S1*C*). Among Type II genes, *Atoh1* emerged as a top candidate based on its high P0/P63 expression ratio (*SI Appendix*, Fig. S1*B*). Atoh1 is well established as a key regulator of GNP maintenance and proliferation and is predominantly expressed in the cerebellum during developmental stages that closely parallel those of *Tox3* expression (Fig. 1*D* and *SI Appendix*, Fig. S1*C*). Notably, the temporal overlap in the expression of *Tox3*/*TOX3* and *Atoh1*/*ATOH1* coincides with the critical phases of GNP generation, migration, and expansion in the developing cerebellum.

To further validate the RNA-seq results and examine the spatial-temporal expression of *Tox3* in different organs, we generated a transgenic mouse model with a *lacZ* reporter gene inserted into the *Tox3* genomic locus (Fig. 1*E* and *SI Appendix*, Fig. S1*D*). X-gal staining results demonstrated high expression of *Tox3* in the brain and spinal cord at E12.5 (Fig. 1*F*). *Tox3* is highly expressed in the cerebellum as well as olfactory bulb and corpus callosum at P1 (Fig. 1*F*). At P7, *Tox3* expression remained detectable in both the cerebellum and the olfactory bulb (*SI Appendix*, Fig. S1*E*). *Tox3* expression was also detectable in the kidneys at P7 but was not observed in the liver, spleen, and lung (*SI Appendix*, Fig. S1*F*). Overall, the X-gal staining results were consistent with the RNA-seq data, both indicating robust *Tox3* expression in the brain, cerebellum, and kidneys during the perinatal period, with decreased expression observed postnatally.

Given the potential role of Tox3 in the developmental proliferation of cerebellar GNPs and its association with medulloblastoma, a cancer that can arise from uncontrolled proliferation of these progenitors, we conducted further analysis. Specifically, we performed immunohistochemical staining on P1 mouse cerebellum sections using an anti-Tox3 antibody and the Purkinje cell marker Calbindin. This confirmed the expression of Tox3 protein in the external granule layer and Purkinje cells. We validated the antibody specificity using the *Nestin-Cre;Tox3^f/f^* mutant tissue which deletes *Tox3* in all neural cell types (Fig. 1*G*).

### Mice with *Tox3* Deficiency Show Severe Ataxia and Cerebellar Hypoplasia.

**Fig. 2.**
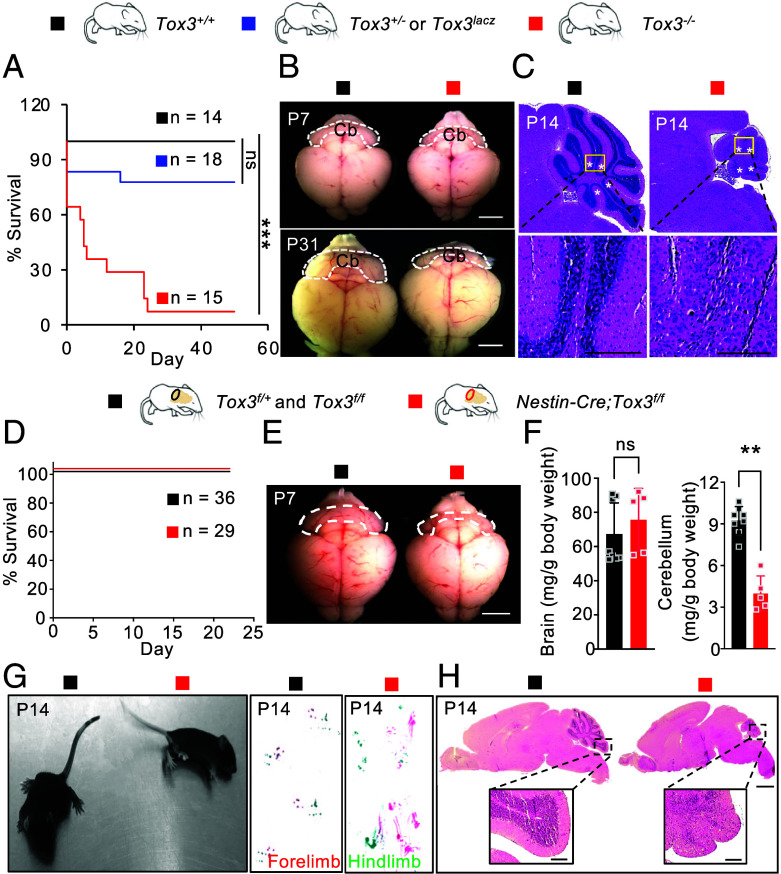
Germline and neural deletion of *Tox3* leads to severe ataxia and cerebellar hypoplasia. (*A*) Kaplan–Meier survival analysis of *Tox3* global knockout mice (*Tox3^+/+^,* n = 14;*Tox3^+/−^*, n = 18; *Tox3^−/−^*, n = 15). (*B*) Whole-mount images of brains from control and *Tox3* knockout mice at P7 and P31. Cb, cerebellum. (*C*) Representative H&E-stained cerebellar sections from control and knockout mice at P14. The four principal fissures (asterisks) and five cardinal lobes remain evident in the knockout cerebellum. (*D*) Kaplan–Meier survival analysis of conditional knockout mice (*Tox3^f/+^* and *Tox3^f/f^*, n = 36; *Nestin-Cre;Tox3^f/f^*, n = 29). (*E*) Gross morphology of brains from control and mutant mice at P7. (*F*) Quantification of brain and cerebellum weights in control and mutant mice at P7 (*Tox3^f/+^*and *Tox3^f/f^*, n = 8; *Nestin-Cre;Tox3^f/f^*, n = 5). (*G*) Representative images illustrating ataxia in mutant mice and footprint patterns of control and mutant mice at P14. (*H*) Representative H&E-stained cerebellar sections from control and mutant mice at P14. Values are reported as mean ± SEM. ns, not significant; **P < 0.01; ***P < 0.001. [Scale bar: 2 mm (*B*), 500 μm (*C*), 2 mm (*E*), 500 μm (*H*), 100 μm (*H*, insert).]

To investigate the physiological function of *Tox3*, we generated mice with germline inactivation of *Tox3*. Homozygous *Tox3^−/−^* mice were born at the expected Mendelian frequency. However, more than 70% of these mice died within 12 d after birth (Fig. 2*A*). Among the survivors, notable differences in size and body weight were observed, with the knockout animals displaying a markedly smaller stature (*SI Appendix*, Fig. S2 *A* and *B*). Notably, these knockout animals exhibited significant motor impairments, characterized by tremors and a lack of balance, suggestive of ataxic conditions (Movie S1; knockout mice are smaller). Subsequent examination of the cerebella at P7 revealed a noticeable reduction in size compared to the control group, with the disparity becoming more pronounced by P31 (Fig. 2*B*). Hematoxylin and eosin (H&E) staining was performed to further investigate the effects of *Tox3* inactivation on organs exhibiting high *Tox3* expression. Comparing midsagittal sections, both *Tox3^−/−^* cerebella and brains appeared smaller at P14 (*SI Appendix*, Fig. S2*C*). A striking difference in the cytoarchitecture of the cerebella between the control and knockout groups was observed, with the knockout group exhibiting severe hypoplasia and an almost complete absence of the internal granule cell layer (Fig. 2*C*). However, foliation was less affected, as the five cardinal lobes, delineated by the four principal fissures, were still evident in the knockout cerebellum. No overt histological differences were observed in the brain, including the hippocampus (insert, *SI Appendix*, Fig. S2*C*). Kidney micrographs showed that, aside from the smaller size of the knockout kidneys, no obvious histological differences were observed (*SI Appendix*, Fig. S2*D*). Similarly, examination of the heart micrographs did not reveal any obvious pathological alterations in the knockout heart at P14 (*SI Appendix*, Fig. S2*E*). In summary, germline inactivation of *Tox3* results in lethality, ataxia, and a striking failure of postnatal cerebellar expansion. Importantly, early cerebellar patterning and foliation were relatively preserved, indicating that *Tox3* is dispensable for initial lineage specification but is essential for sustaining progenitor output during postnatal cerebellar growth.

Because *Tox3* is highly expressed in GNPs which are crucial for cerebellar development and motor control, we next generated mice lacking *Tox3* in the nervous system (*Nestin-Cre;Tox3^f/f^*). Knockout efficiency was confirmed by reverse transcription coupled with qPCR (RT-qPCR) analysis of P1 cerebella, showing near-complete deletion of *Tox3* in mutants (98 ± 1.6%; *SI Appendix*, Fig. S2*F*). In contrast to global knockout mice, no neonatal deaths were observed in conditional knockout mice (Fig. 2*D*). However, mutant mice with neural deletion of *Tox3* also displayed smaller body sizes (*SI Appendix*, Fig. S2 *G* and *H*), reduced cerebellum sizes (Fig. 2 *E* and *F*), and severe ataxia (Fig. 2*G*). Motor deficits first became apparent around P10 (Movie S2, mutant on the right) and progressed in severity by P14 (Fig. 2*G* and Movie S3, mutant on the right). Histological examination at P14 unveiled severe cerebellar hypoplasia, characterized by a near absence of granule cells (Fig. 2*H*). Intriguingly, although *Tox3* is expressed during early development in both the brain and cerebellum (Fig. 1 *D* and *F*), the relative weight of the cerebellum but not the whole brain after normalization to the body weight is selectively impaired in *Tox3* mutant mice (Fig. 2*F*). This result suggests that *Tox3* is either not required or there is a redundant mechanism for the development of the rest of the brain. Altogether, these findings underscore a critical role for *Tox3* in cerebellar development, with its targeted inactivation leading to cerebellar hypoplasia and motor deficits in mutant mice.

### Inactivation of *Tox3* Causes Profound Defects in Cerebellar GNPs.

**Fig. 3.**
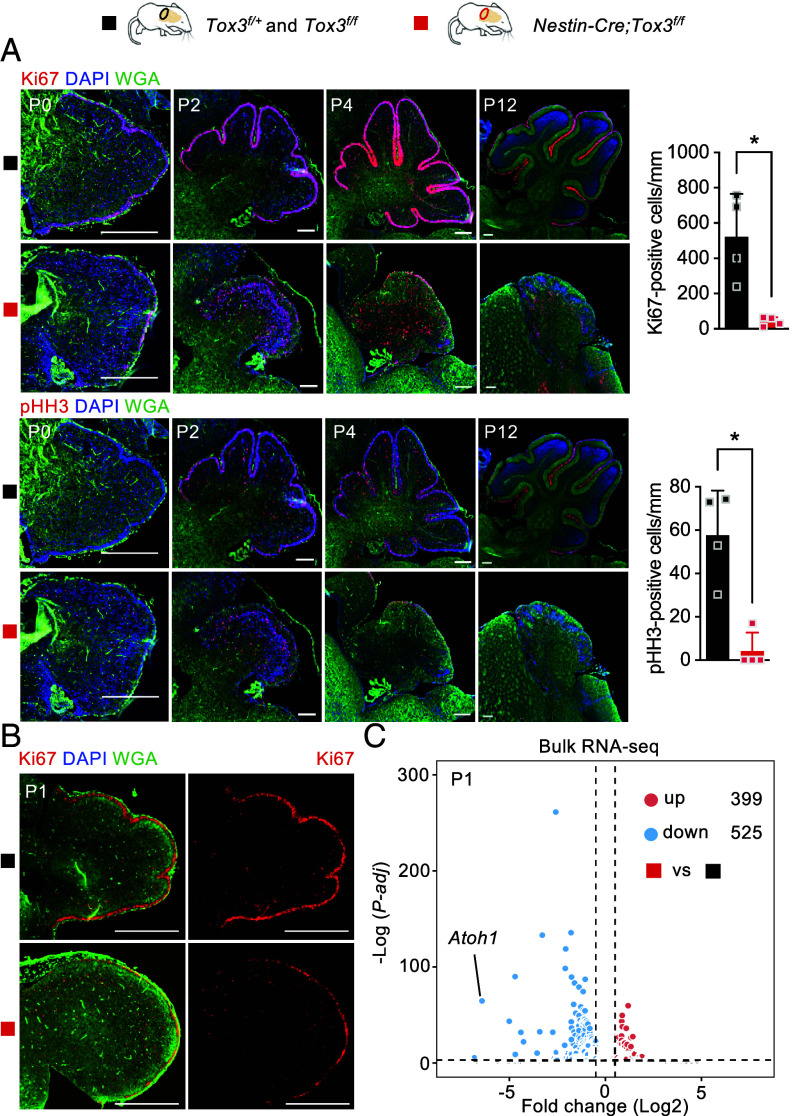
Loss of *Tox3* causes profound defects in cerebellar GNPs. (*A*) Representative images of Ki67 and pHH3 staining in control and mutant cerebella at P0, P2, P4, and P12. Quantification of proliferating cells in the P2–P4 cerebellum is shown on the *Right*. pHH3, phospho-histone 3 (*Tox3^f/+^*and *Tox3^f/f^*, n = 4 mice; *Nestin-Cre;Tox3^f/f^*, n = 4 mice). EGL, external granule layer. (*B*) Ki67 staining of control and mutant cerebella at P1. (*C*) Volcano plot showing differentially expressed genes in mutant cerebella identified by bulk RNA-seq analysis. Values are reported as mean ± SEM. *P < 0.05. [Scale bar: 500 μm (*A* and *B*).]

To determine whether the observed cerebellar hypoplasia stemmed from impaired proliferation of GNPs, we evaluated Ki67- and phosphorylated histone H3 (pHH3)-positive cells in the external granule layer (Fig. 3*A* and *SI Appendix*, Fig. S3*A*). At P0, the cerebellar size of the mutant and control littermates was comparable, with a substantial number of Ki67- and pHH3-positive cells detected in the external granule layer of the mutant mice. This indicates that the proliferation deficiency mainly occurred during postnatal development. By P2, a substantial number of Ki67- and pHH3-positive cells were observed in the external granule cell of control cerebella. However, only a sparse population of Ki67- and pHH3-positive cells persisted in the external granule layer of mutant mice. Notably, between P2 and P4, the number of Ki67- and pHH3-positive external granule cells was significantly reduced by >90% in *Nestin-Cre;Tox3^f/f^* mice (Fig. 3*A*). By P12, a proliferating layer of GNPs remained evident in the external granule layer of control cerebella, a feature that was absent in the mutants. These findings substantiate that inactivation of *Tox3* leads to impaired proliferation of GNPs during critical stages of cerebellar development. Moreover, a scattering of Purkinje cells was also observed in the mutant cerebellum (*SI Appendix*, Fig. S3*B*). We also observed an abnormal excess of proliferative and mitotic cells in the inner layer of the mutant cerebellum at P2, which was absent in control animals (Fig. 3*A*). We further examined the phenotype at P1 and observed normal localization but visually reduced proliferation of GNPs (Fig. 3*B*). These results suggest reductions in either the number of GNPs, the proliferation rate of GNPs, or both on P1 mutant cerebella. The presence of mitotic cells in the P2 mutant cerebellum may reflect mislocalization or premature migration of proliferative GNPs from the external granule layer into the internal cerebellar regions. These abnormalities likely contribute to the progressive development of the ataxic phenotype.

To comprehensively assess Tox3-mediated transcriptional changes, we performed bulk RNA-seq analysis of P1 cerebella, identifying 525 downregulated and 399 upregulated genes in mutant samples. Notably, among the downregulated genes, *Atoh1* emerged as one of the most significantly affected (Fig. 3*C*). Atoh1 has been reported to be essential for maintaining GNPs in an immature proliferative state by regulating primary cilia formation (22, 23). Furthermore, GO analysis of the downregulated genes highlighted that the top 10 enriched pathways were primarily associated with cell proliferation, and the top 10 non-cell-cycle-related pathways were related to neuron differentiation and cerebellum development (*SI Appendix*, Fig. S3*C*). Specifically, analysis of 43 S phase and 54 G2/M phase cell cycle-promoting genes revealed pronounced downregulation in mutant cerebella (*SI Appendix*, Fig. S3*D*). RT-qPCR analysis of seven selected cell cycle genes using an independent sample set corroborated their downregulation (*SI Appendix*, Fig. S3*E*). Additionally, assessment of the primary cilia demonstrated that *Tox3* inactivation resulted in reductions in both the number of ciliated cells and the average cilia length among the ciliated cells (*SI Appendix*, Fig. S3*F*). Together, these findings underscore an indispensable role for Tox3 in maintaining Atoh1-dependent transcriptional program in GNPs during postnatal cerebellar development. Rather than simply promoting cell cycle progression, Tox3 appears to stabilize progenitor identity by sustaining the expression of *Atoh1* and its downstream gene network.

### Single-Nucleus RNA-Sequencing Analysis (snRNA-seq) Reveals Transcriptional Dynamics at Single-Cell Resolution.

**Fig. 4.**
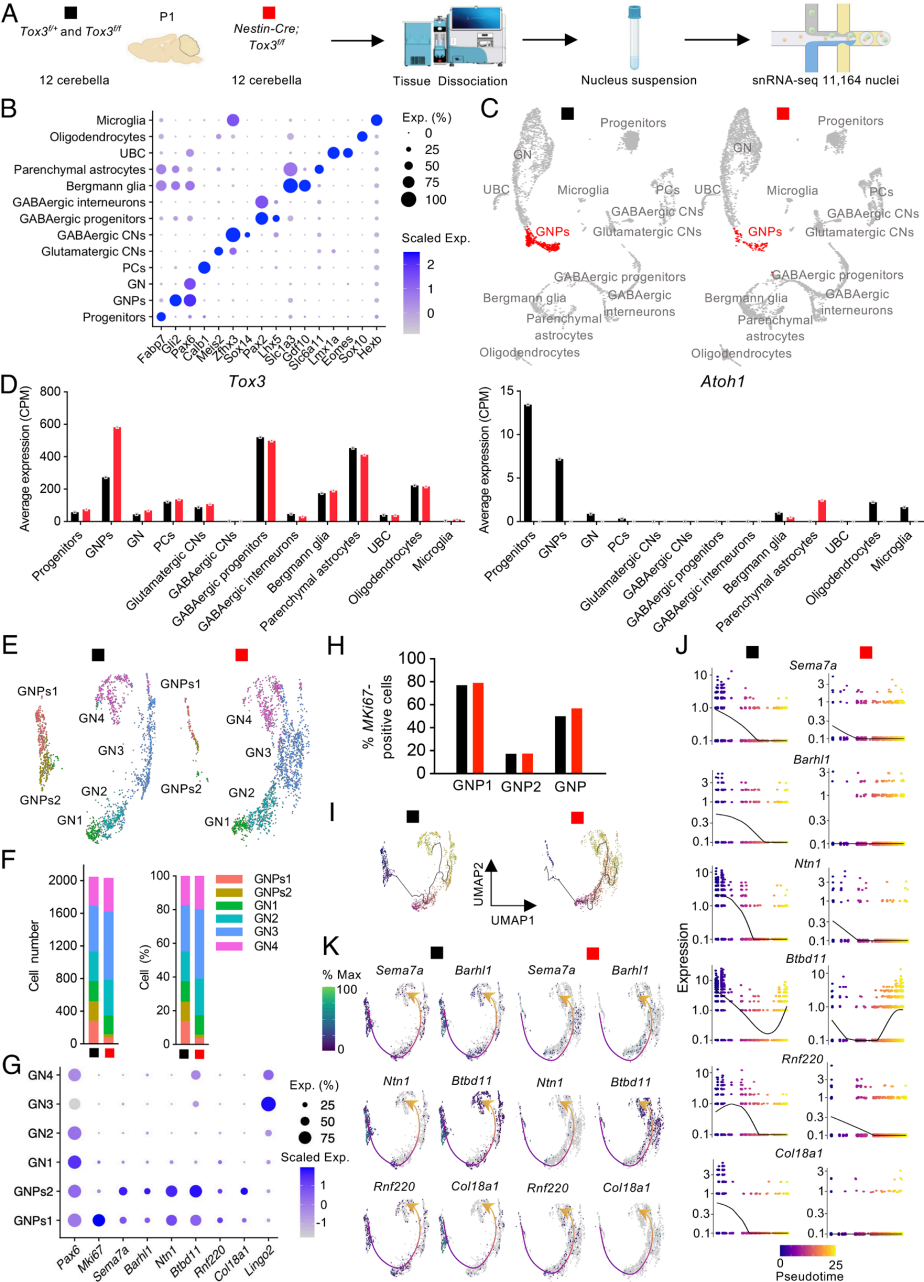
snRNA-seq reveals that *Tox3* deficiency impairs the maintenance of GNPs in the neonatal cerebellum. (*A*) Schematic of the experimental design for snRNA-seq analyses. Postnatal day 1 (P1) cerebella were collected, and cerebella from 12 control and 12 mutant mice were pooled to generate one control and one mutant sequencing library for comparison. (*B*) Dot plots showing the expression of marker genes for each cluster. GNPs, granule neuron progenitors; GN, granule neuron; PCs, Purkinje cells; CNs, cerebellar nuclear neurons; UBC, unipolar brush cell. (*C*) UMAP plots showing single-nucleus transcriptomes from control and mutant cerebella. The major difference between the two groups is the number of GNPs, highlighted in red. (*D*) snRNA-seq showing the expression of *Tox3* and *Atoh1* across different cell types. (*E*) UMAP plots of GNPs and GNs, which were further subdivided into six distinct clusters. (*F*) Cell numbers and proportions of each GN cluster in each snRNA-seq sample. (*G*) Dot plots showing expression of marker genes and *Tox3* in each GNP and GN cluster. (*H*) Comparable numbers of Ki67-positive GNPs in control and mutant mice. (*I*) Monocle3 pseudotime analysis of the six clusters depicting the differentiation trajectory from GNPs1 to GN4. (*J*) Pseudotime-ordered gene expression for GNP-enriched genes (*Sema7a*, *Barhl1*, *Ntn1*, *Btbd11*, *Rnf220*, and *Col18a1*). Cells are colored by pseudotime values. (*K*) UMAP showing the expression of *Sema7a*, *Barhl1*, *Ntn1*, *Btbd11*, *Rnf220*, and *Col18a1* along the GNPs1-to-GN4 differentiation trajectory. Cells are colored by normalized expression levels, and arrowed curves indicate the pseudotime trajectory.

To profile the response to *Tox3* inactivation at single-cell resolution, we performed snRNA-seq on cerebella from control and mutant mice at P1. To ensure robustness and account for potential variability, we dissected 12 cerebella from each group. We captured 11,164 single nuclei from both samples with high integrity and sequenced a median of 2,095 genes per nucleus (Fig. 4*A* and *SI Appendix*, Fig. S4*A*). Integration of datasets from both groups enabled cell-type clustering for cross-sample comparison (24, 25), identifying a total of 13 clusters based on top differentially expressed genes and known cellular marker genes (Fig. 4 *B* and *C* and *SI Appendix*, Fig. S4 *B*–*D*).

*Tox3* expression waspredominantly observed in GNPs, Purkinje cells, glutamatergic cerebellar nuclear neurons (CNs), GABAergic progenitors, Bergmann glia, parenchymal astrocytes, and oligodendrocytes (Fig. 4*D*). *Atoh1* expression was confined to control progenitor cells and GNPs and was almost absent in mutants (Fig. 4*D*). Consistent with the reported role of *Atoh1* in maintaining GNPs, we observed a dramatic reduction in the percentage of GNPs from 11.2% in control cerebella to 1.8% in mutant cerebella (Fig. 4*C* and *SI Appendix*, Fig. S4*E*). Given comparable numbers of total nuclei as well as GNs in the control and mutant samples (*SI Appendix*, Fig. S4*E*), the observed selective reduction of GNPs in mutant mice is likely due to biological differences rather than technical artifacts.

### Tox3 Is Essential for the Maintaining of GNPs.

To gain more insights into the loss of GNPs in the mutant cerebellum, we isolated GNP and GN clusters from the snRNA-seq dataset for further analysis (Fig. 4*E*). Nuclei were grouped into six clusters, with 2,050 nuclei identified in the control and 2,035 nuclei in the mutant samples. Notably, the proportion of GNPs in the mutant, represented by clusters GNPs1 and GNPs2, decreased markedly from 25.3 to 5.7%, accompanied by the expansion of the granular neuron population 3 (GN3) (Fig. 4 *E* and *F*). Gene expression analysis revealed that the granule cell marker gene *Pax6* was expressed across all clusters (Fig. 4*G*). *Mki67* was preferentially expressed in clusters GNPs1 and downregulated in GNPs2 (Fig. 4*G*). GN3 was marked by the *Lingo2-*high signature.

The reduction of GNPs in the *Tox3* mutant cerebellum could be due to the decrease of GNP proliferation, premature differentiation of GNPs, or both. To distinguish among these possibilities, we first examined the proliferation rate of GNPs in control and mutant animals and observed comparable Ki67-positive GNP1s (Fig. 4*H*), the major proliferative population (Fig. 4*G*), suggesting that the proliferation of *Tox3*-deficient GNPs is largely normal. The expansion of GNs in mutant cerebella, particularly the GN3 population, suggested premature differentiation. To further characterize the differentiation trajectory, we performed a pseudotime analysis using Monocle (Fig. 4*I*). Differential gene analysis identified 105 upregulated genes and 49 downregulated genes along the differentiation trajectory from GNPs1 to GN4 (*SI Appendix*, Fig. S5*A*). Downregulated genes were enriched for nuclear division and chromosome segregation, consistent with cell cycle exit, whereas upregulated genes were associated with synaptic assembly, organization, and regulation of synapse structure or activity, indicative of GN differentiation (*SI Appendix*, Fig. S5*B*). These results indicate that pseudotime analysis captures the differentiation trajectory of GNPs and enables assessment of gene expression dynamics during this process. We next examined the expression of genes that are enriched in GNPs (*Sema7a, Barhl1, Ntn1, Btbd11, Rnf220, and Col18a1*) (Fig. 4*G*) along the differentiation trajectory using pseudotime rank-ordered gene expression and Uniform Manifold Approximation and Projection (UMAP) (Fig. 4 *J* and *K*). Remarkably, the number of cells expressing these six genes were dramatically decreased in mutants, suggesting early depletion and premature differentiation of GNPs in animal lacking *Tox3*. Thus, single cell transcriptomic analyses reveal that *Tox3* deficiency primarily impairs the maintenance of GNPs rather than their intrinsic proliferative capacity. These data indicate that Tox3 functions to preserve progenitor state fidelity and delay premature differentiation, highlighting a role in transcriptional stability rather than direct cell cycle control.

### Tox3 Plays a Pivotal Role in Medulloblastoma.

**Fig. 5.**
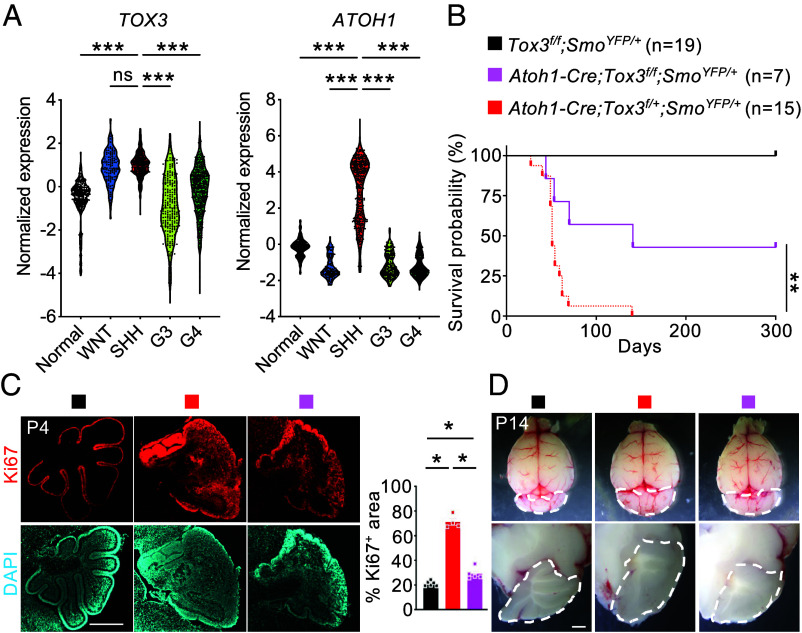
Expression and function of *Tox3* in medulloblastoma. (*A*) Violin plots showing the distribution of *TOX3* and *ATOH1* expression across molecular subgroups (WNT, SHH, Group 3, and Group 4) in 23 independent transcriptomic datasets (GSE124814), comprising 1,350 medulloblastoma samples and 291 normal brain samples. (*B*) Kaplan–Meier survival curves of animals with the indicated genotypes. (*C*) Immunostaining of cerebellar sections showing proliferating (Ki67-positive) cells at postnatal day 4 (P4). (*D*) Whole-mount and cross-sectional views of cerebella from the indicated genotypes at P14. Dashed lines outline the cerebellum. Values are reported as mean ± SEM. ns, not significant; *P < 0.05, **P < 0.01, ***P < 0.001. [Scale bar: 500 μm (*C*), 2 mm (*D*).]

Uncontrolled *Atoh1*-positive cerebellar GNPs can give rise to medulloblastoma, and deletion of *Atoh1* prevents Shh-induced neoplasm in a mouse model (22). Given that *Tox3* deficiency causes severe loss of *Atoh1* expression and depletion of GNPs, we hypothesized that *Tox3* deletion may also prevent Shh-driven medulloblastoma. To explore this, we first analyzed *TOX3* and *ATOH1* expression across molecular subgroups of human medulloblastoma using twenty-three published transcription datasets (26). *TOX3* was highly expressed in group I (WNT) and group II (SHH) medulloblastomas, moderately expressed in group IV, and low in group III tumors (Fig. 5*A*). As expected, *ATOH1* was highly expressed in SHH-type medulloblastoma (Fig. 5*A*). We then assessed the functional importance of *Tox3* is Shh-driven medulloblastoma using the *Atoh1-Cre;Rosa26^SmoM2/+^* mouse model, in which constitutively active Smoothened drives Gli-dependent gene expression in Atoh1-positive cells (27). Animals with 1 copy of the *Tox3* allele (*Atoh1-Cre;Rosa26^SmoM2/+^;Tox3^f/+^*) showed a median survival similar to that of previously reported control animals (*Atoh1-Cre;Rosa26^SmoM2/+^*) (51 vs. 52 d) (28). But homozygous genetic deletion of *Tox3* (*Atoh1-Cre;Rosa26^SmoM2/+^;Tox3^f/f^*) significantly prolonged median survival (141 d, Fig. 5*B*). Further analyses at earlier time points demonstrated reduced cell proliferation (Fig. 5*C*) and smaller cerebellar size (Fig. 5*D*) in mice homozygous for *Tox3* deletion. Together, these data suggest that Tox3 may represent a potential therapeutic target for SHH-driven medulloblastoma.

### Tox3 and Atoh1 Share a Substantial Number of Direct Target Genes and Physically Interact with Each Other.

**Fig. 6.**
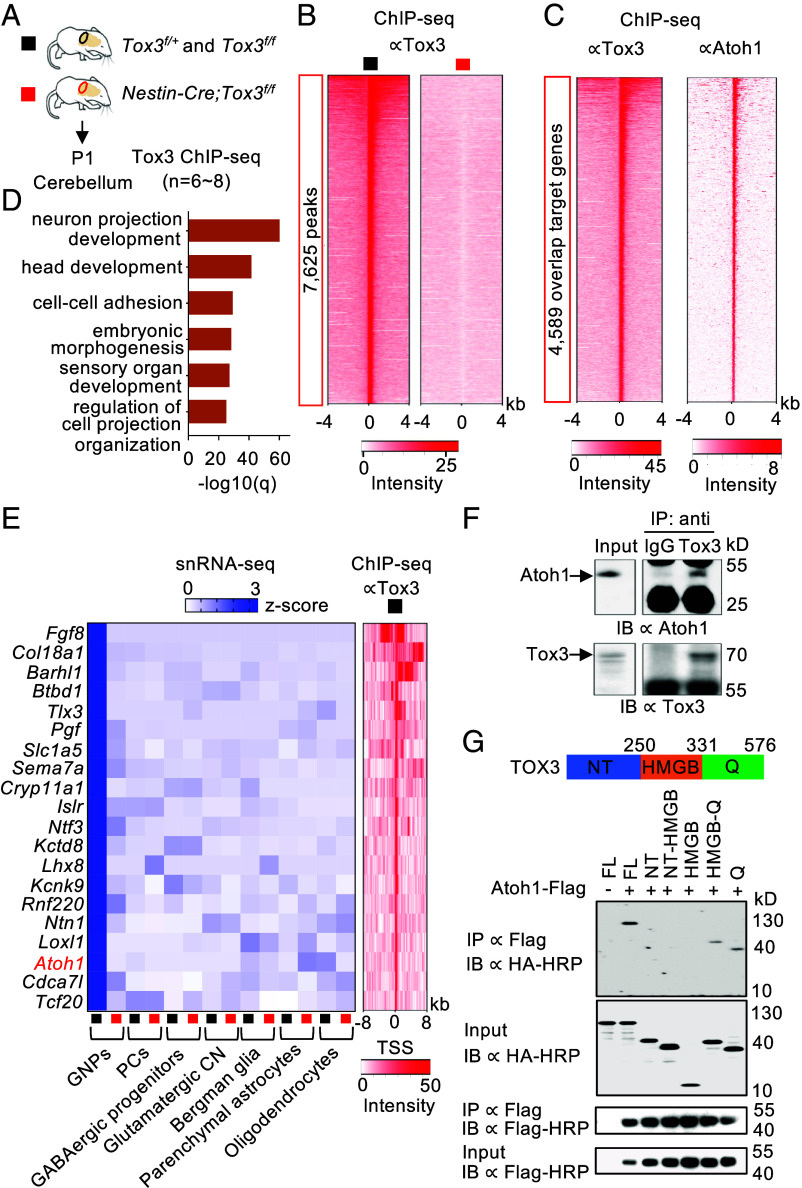
Tox3 and Atoh1 share a substantial number of direct target genes and physically interact. (*A*) Schematic showing cerebellar tissue collection for ChIP-seq. (*B*) Heatmap showing ChIP-seq signal enrichment around Tox3 binding sites. TSS, transcription start site. (*C*) Heatmap showing ChIP-seq signal enrichment around genes cotargeted by Tox3 and Atoh1. (*D*) Top six enriched terms among shared Tox3 and Atoh1 target genes. (*E*) Heatmap showing that genes predominantly expressed in GNPs are downregulated and directly targeted by Tox3. PCs, Purkinje cells; CN, cerebellar nuclear neuron. (*F*) Coimmunoprecipitation of endogenous Tox3 and Atoh1 proteins from postnatal day 7 mouse cerebella. IP, immunoprecipitation; IB, immunoblotting. (*G*) Coimmunoprecipitation of TOX3 with Atoh1 proteins following overexpression in HEK293T cells. A schematic of TOX3 protein domains is shown at the *Top*. FL, full-length; NT, N-terminal; Q, glutamine-rich repeats.

To identify Tox3 direct target genes, we performed chromatin immunoprecipitation coupled with sequencing (ChIP-seq) analysis using P1 cerebella (Fig. 6*A*), which revealed the distribution and density of Tox3 binding peaks in the genome (*SI Appendix*, Fig. S6*A*). Compared to the analysis using mutant cerebella, the Tox3 ChIP-seq using control cerebella identified 7,625 differential peaks (Fig. 6*B*). Remarkably, 60% of Tox3 binding peaks overlapped with or were located near Atoh1 binding sites in the neonatal mouse cerebellum, based on a published Atoh1 ChIP-seq dataset (Fig. 6*C*). GO analysis of these shared targets revealed significant enrichment for pathways involved in neuron projection development, head development, cell–cell adhesion, embryonic morphogenesis, sensory organ development, and regulation of cell projection organization (Fig. 6*D*). These findings indicate that Tox3 and Atoh1 share closely related target genes and functions during cerebellar development. Furthermore, GO analysis of genes nearest to the translation start site of Tox3 and Atoh1 binding peaks identified neuron projection development and head development among the top six shared enriched pathways (*SI Appendix*, Fig. S6*B*). These results suggest that Tox3 and Atoh1 share numerous direct target genes involved in neural development.

In cells with high *Tox3* expression, the top 20 GNP-enriched genes were all downregulated in *Tox3* mutant cerebella and are direct targets of Tox3 (Fig. 6*E*). Notably, *Atoh1*, one of the most significantly downregulated genes in *Tox3* mutants (Fig. 3*C*), is also a direct Tox3 target.

Because many Tox3 and Atoh1 binding peaks overlap, we hypothesized that Tox3 physically interacts with Atoh1. To test this, we performed coimmunoprecipitation using protein lysates from P7 mouse cerebella. Indeed, Atoh1 was detected in complexes immunoprecipitated with an anti-Tox3 antibody (Fig. 6*F*). We further confirmed that TOX3 and Atoh1 coimmunoprecipitate when heterologously expressed in HEK293T cells (Fig. 6*G*). Truncation analysis revealed that the polyglutamine-rich (polyQ) domain of TOX3 is necessary for binding Atoh1 and, although the polyQ region alone can bind Atoh1, the interaction is weaker than with full-length TOX3, suggesting that additional domains contribute to binding affinity (Fig. 6*G*). Altogether, these findings support that Tox3 and Atoh1 form a protein complex targeting common downstream genes.

### Tox3 Functions as an Atoh1 Coactivator to Induce Target Gene Expression.

**Fig. 7.**
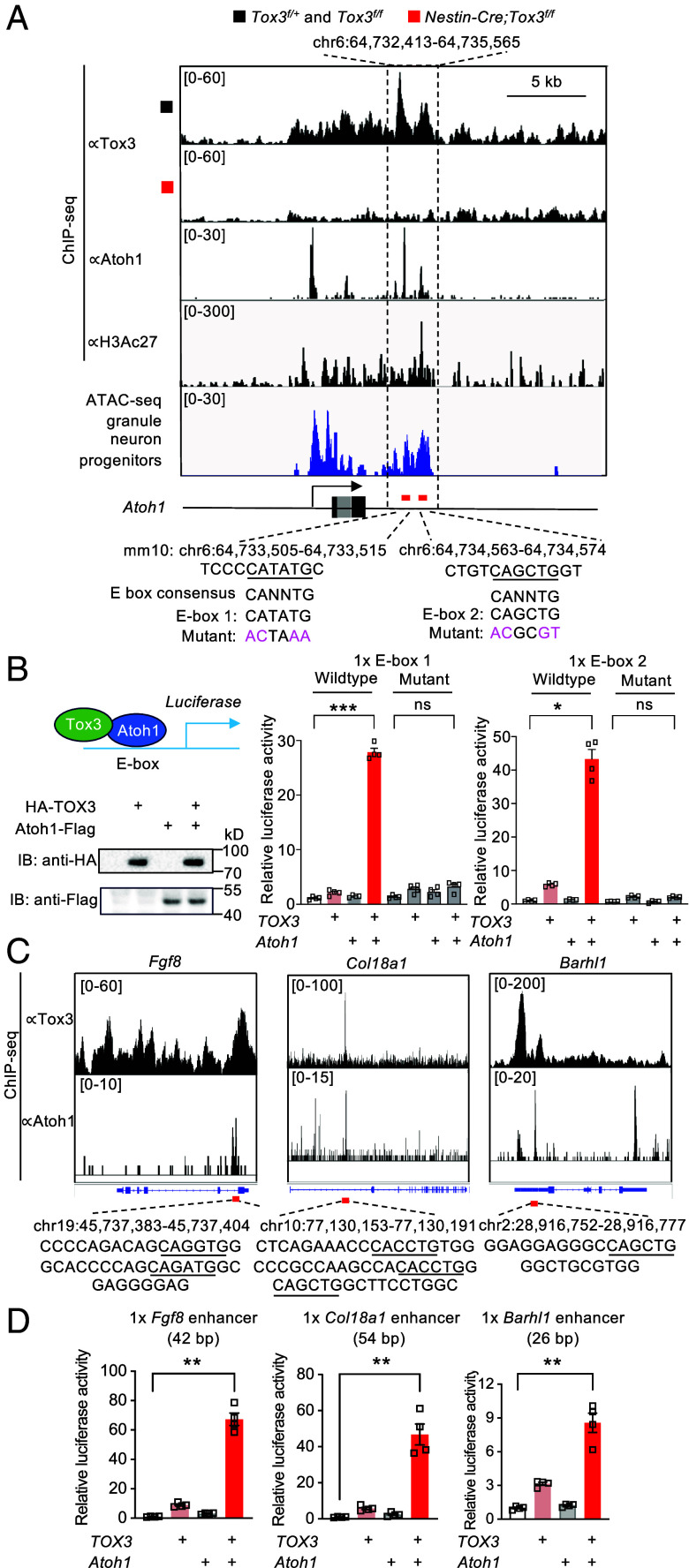
Tox3 and Atoh1 synergistically activate downstream gene enhancers. (*A*) ChIP-seq peaks showing that both Tox3 and Atoh1 bind to a downstream enhancer of *Atoh1*, marked by H3K27ac ChIP-seq. ATAC-seq indicates that this enhancer is accessible to transcription factors in GNPs. The sequences of Atoh1 E-boxes and their mutated counterparts are shown. (*B*) Luciferase assays demonstrating that Tox3 and Atoh1 synergistically activate both *Atoh1* 3’ E-boxes, whereas mutation of the E-boxes abolishes enhancer activity. Comparable expression levels of TOX3 and Atoh1 when expressed individually or in combination were confirmed by immunoblotting. (*C*) ChIP-seq peaks for Tox3 and Atoh1, together with E-box sequences, at enhancers of three additional direct target genes: *Fgf8*, *Col18a1,* and *Barhl1*. (*D*) Luciferase assays showing synergistic activation of *Fgf8*, *Col18a1*, and *Barhl1* enhancers by Tox3 and Atoh1. Values are reported as mean ± SEM. For luciferase assays in panel *B*, a single E-box copy was inserted into the reporter plasmid; for panel *D*, a single copy of the enhancer shown in panel *C* was used. Data are representative of three independent experiments. ns, not significant; *P < 0.05, **P < 0.01, ***P < 0.001.

ChIP-seq analysis revealed that Tox3 and Atoh1 bind near a distal *Atoh1* enhancer marked by H3Ac27, which is accessible in GNPs, as shown by ATAC-seq peaks (Fig. 7*A*). Two Atoh1 binding E-boxes (E-box1 and E-box2) lie adjacent to Tox3 binding peaks within this region (Fig. 7*A*). Remarkably, deletion of this enhancer in mice impairs balance and cerebellar development (29), similar to *Tox3* mutant phenotypes (Fig. 2 *G* and *H*), suggesting that these E-boxes are critical for Tox3-Atoh1-mediated transactivation of *Atoh1*. To test this, one copy of the E-box sequence was inserted into a luciferase reporter, and reporter activity was measured in HEK293T cells coexpressing TOX3 and/or Atoh1 (Fig. 7*B*). Expression of TOX3 or Atoh1 alone induced minimal activity, whereas coexpression yielded a striking >25-fold synergistic induction, which was almost completely abolished by E-box mutations (Fig. 7*B*). Since Tox3 does not directly bind E boxes, these results suggest that Tox3 functions as an Atoh1 cofactor in an E box-dependent manner.

To assess the generality of Tox3 and Atoh1 synergy in driving downstream gene expression, we examined three top GNP-enriched target genes: *Fgf8*, *Col18a1,* and *Barhl1* (Fig. 6*E*). Atoh1 E-boxes bound by both Tox3 and Atoh1 in proximity were identified (Fig. 7*C*). Each enhancer region enclosing the E-box was cloned into a luciferase reporter construct. Strong luciferase induction was observed for all three reporters only when both TOX3 and Atoh1 were coexpressed (Fig. 7*D*), supporting Tox3 as an Atoh1 cofactor in transactivating downstream targets.

To assess the role of Tox3 and Atoh1 in regulating GNP-enriched genes, we compared RNA-seq data from *Tox3* mutant cerebella with published RNA-seq of E18 cerebella lacking *Atoh1* (13). First, *Tox3* expression was unaffected in *Atoh1*-deficient cerebella (*SI Appendix*, Fig. S6*C*), indicating that *Atoh1* is not required to maintain *Tox3* expression at this stage. Second, among the top 19 downregulated GNP-enriched genes in *Tox3* mutants (Fig. 6*E*), six genes–*Pgf*, *Cyp11a1*, *Kctd8*, *Rnf220*, *Ntn1,* and *Tcf20*–were not altered in *Atoh1* mutants (*SI Appendix*, Fig. S6*D*), suggesting that Tox3 can activate these genes independently of Atoh1. Third, 13 genes were significantly downregulated in *Atoh1* mutants (*SI Appendix*, Fig. S6*E*). Notably, Tox3 and Atoh1 binding peaks overlapped at six of these loci–*Slc1a5*, *Sema7a*, *Atoh1*, *Fgf8*, *Col18a1,* and *Barhl1* (*SI Appendix*, Fig. S6*F* and Fig. 7*C*)–indicating that Tox3 likely functions as an Atoh1 coactivator. For the remaining seven genes, no overlapping binding peaks were detected (*SI Appendix*, Fig. S6*G*), suggesting that their downregulation in *Tox3* mutants may result from secondary loss of Atoh1. Collectively, these results support that Tox3 regulates downstream gene expression through both Atoh1-dependent and -independent mechanisms.

### Tox3 as a Putative Regulator of Cerebellum Expansion during Vertebrate Evolution.

Given the evolutionary importance of GNP proliferation in cerebellar expansion, and the essential role of Tox3 in this process during mouse development, we performed ortholog and protein structural similarity analyses of Tox3. Tox3 orthologs were identified in cartilaginous fishes (e.g., the small-spotted catshark)–the earliest vertebrates known to possess a well-developed cerebellum (*SI Appendix*, Fig. S7 *A* and *B*). This evolutionary timeline raises the possibility that Tox3 contributed to the emergence or specialization of the vertebrate cerebellum.

To further explore its evolutionary context, we performed phylogenetic analyses of Tox3 and Tox3-like proteins (*SI Appendix*, Fig. S7), as well as Atoh1, atoh1a, and atoh1-like proteins (*SI Appendix*, Fig. S8), extending beyond vertebrates to include Ciona (an invertebrate chordate, the closest living relative of vertebrates) and Trichoplax (a basal metazoan lacking neurons but exhibiting neurotransmitter signaling). Sequence alignment showed that, relative to the mouse Tox3, the Trichoplax Tox3-like protein exhibited the lowest similarity, followed by those from Ciona and sea lamprey. From cartilaginous fishes onward, the region surrounding HMG-box domain of Tox3 is highly conserved, consistent with a role in cerebellar evolution. In contrast, Atoh1 and atoh1-like proteins harbor a highly conserved bHLH domain (*SI Appendix*, Fig. S8), indicating that Atoh1 function is evolutionarily older and broadly conserved across metazoans.

**Fig. 8.**
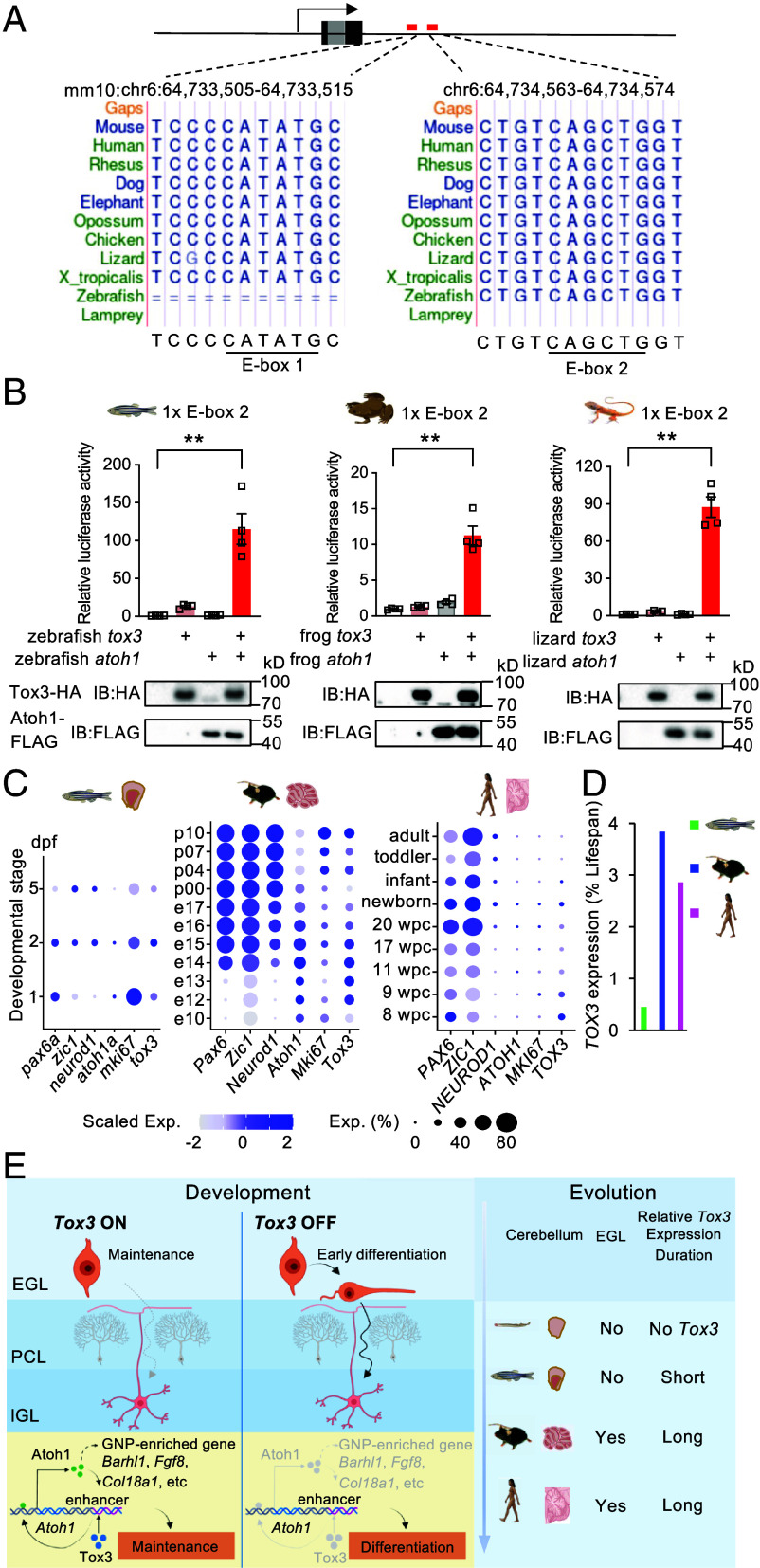
Evolutionarily conserved role of Tox3 as an Atoh1 coactivator and its implication in cerebellar expansion during vertebrate evolution. (*A*) Sequence alignments showing the conservation of both *Atoh1 3’* E-box sequences. In particular, E-box 2 is ultraconserved across vertebrate species including zebrafish, *X. tropicalis*, lizard, and mammals. (*B*) Luciferase assays showing that zebrafish, *X. tropicalis*, and lizard Tox3 and Atoh1 proteins synergistically activate *Atoh1* 3’ E-box 2. (*C*) Single-cell RNA-seq analyses showing that the duration of *Tox3* expression in the cerebellum correlates with the duration of GNP proliferation and cerebellar complexity across species. Dot plots display expression of granule cell marker genes together with *Mki67/mki67*, *Atoh1/atoh1a*, and *Tox3/tox3* across developmental stages in granule cell cluster from zebrafish, mouse, and human single-cell RNA-seq datasets. dpf, days postfertilization; wpc, weeks postconception. (*D*) Comparison of *Tox3* expression duration in the cerebellum relative to the average lifespan of zebrafish, mouse, and human. (*E*) Proposed model illustrating the role of Tox3 in GNP maintenance during cerebellar development and vertebrate evolution. For luciferase assays, a single copy of the E-box was inserted into the reporter plasmid. Luciferase assay results are representative of three independent experiments. Values are reported as mean ± SEM. **P < 0.01. EGL, External granule layer; IGL, Internal granule layer; PCL, Purkinje cell layer; GNP, granule neuron progenitor.

Moreover, the downstream enhancer of *Atoh1*, especially the second E-box, is conserved across fish, frogs, lizards, and mammals, whereas both E-boxes are absent in lamprey (Fig. 8*A*). To test whether Tox3 in these species can similarly transactivate *Atoh1* via the conserved E-box, we coexpressed zebrafish, frog, and lizard *Tox3* and *Atoh1*, either individually or in combination, in HEK293T cells and performed luciferase assays. Coexpression of *Tox3* and *Atoh1* robustly activated E-box2-driven reporters, resulting in 115-fold, 11-fold, and 88-fold increases in luciferase activity for zebrafish, *Xenopus tropicalis,* and lizard (Fig. 8*B*). In addition, the lizard E-box1 was also transactivated by Tox3 and Atoh1, yielding a 15-fold increase in reporter activity (*SI Appendix*, Fig. S9*A*). Together, these results suggest that the transactivation of *Atoh1* expression by Tox3 and Atoh1 through the two E-boxes is evolutionarily conserved.

To further examine the role of Tox3 in GNP generation and proliferation across vertebrate evolution, we performed de novo analyses of published scRNA-seq datasets from zebrafish (30), mouse (31), and human (32). In zebrafish, *atoh1a/*b expressing precursor cells are highly proliferative at 2 days post fertilization (dpf) and are primarily localized to the rostro-medial cerebellum during early larval development (5, 33). By 2.5 dpf, *atoh1a/b* expression begins to decline, and by 5 dpf, proliferative activity is largely extinguished in the main cerebellar corpus (5). Consistent with these observations, our scRNA-seq analysis revealed robust expression of *atoh1a* and the proliferation marker *mki67* at 1- and 2-dpf, followed by a marked decline by 5 dpf (Fig. 8*C* and *SI Appendix*, Fig. S9*B*). In contrast, *neurod1*, which marks both immature and mature GNs, was induced by 2 dpf, and maintained through 5 dpf, in agreement with prior reports (34, 35).

The expression profile of *atoh1a*, *mki67*, and *neurod1* demonstrate the suitability of the zebrafish scRNA-seq dataset for identifying candidate genes involved in GNP proliferation. Notably, zebrafish *tox3* was expressed at 1- and 2-dpf and was downregulated by 5 dpf (Fig. 8*C*). These results suggest a potential role of *tox3* in zebrafish cerebellar development and GNP proliferation.

We next analyzed a mouse cerebellar scRNA-seq dataset encompassing 11 developmental time points from E10 to P10 (31). GNs and GNPs were identified based on the expression of established marker genes, including *Pax6*, *Zic1* (36), *Neurod1* (34), and *Atoh1* (*SI Appendix*, Fig. S9*C*). Consistent with our bulk RNA-seq results, more than 40% of GNPs expressed both *Tox3* and *Atoh1* between E12 and E17, corresponding to the period during which progenitors are generated in the rhombic lip and migrate to form external granule layer (Fig. 8*C*). Moreover, over 20% of GN/GNPs continued to express *Tox3* and *Atoh1* between P0 and P10, coinciding with the critical proliferative phase of GNPs within the external granule layer. Together, these expression patterns support shared roles for *Tox3* and *Atoh1* in GNP proliferation and cerebellar development in the mouse.

In humans, the external granule layer persists from ~8 wk postconception to ~2 y of age, coinciding with a prolonged period of GNP proliferation and the onset of cerebellar cortical folding during the third trimester of pregnancy (37). This extended presence of the external granule cell layer provides a prolonged window for GNP expansion. Consistent with this developmental timeline, our scRNA-seq analysis revealed peak expression of *ATOH1* and *TOX3* in the newborn cerebellum, with sustained expression of both genes observed between 9 wk postconception and newborn (Fig. 8*C*). Furthermore, in line with the postnatal disappearance of the external granule layer by 2 y of age, *TOX3* expression declined to relatively low levels thereafter. These observations suggest a potential role for *TOX3* in the maintenance of human GNPs.

To account for interspecies differences, we normalized the duration of *Tox3* expression to the average lifespan of each species. Following normalization, the association between prolonged *Tox3* expression and extended GNP proliferation remained evident (Fig. 8*D* and *SI Appendix*, Table S1), reinforcing our conclusions. Collectively, this cross-species single-cell transcriptomic analysis indicates that the duration of *Tox3* expression in GNPs correlates with the proliferative window of these cells and with increasing cerebellar complexity across vertebrate species.

## Discussion

How transcriptional programs achieve both robustness and specificity during periods of rapid progenitor expansion is a central problem in developmental biology. Lineage-defining transcription factors often exhibit limited intrinsic transactivation capacity, yet must drive sustained, high-level gene expression over extended developmental windows. Our study identifies Tox3 as a critical coregulatory component that resolves this paradox by amplifying and stabilizing Atoh1-dependent transcriptional output in GNPs. These findings illuminate a broader principle of transcriptional network logic, in which robust developmental programs emerge through selective engagement of context-specific cofactors.

Although Atoh1 has long been recognized as indispensable for GNP specification and maintenance (22, 23), it is a relatively weak transcriptional activator when acting alone. This paradox has raised the question of how Atoh1 robustly drives gene expression in vivo. Our data demonstrate that Tox3 serves as a network amplifier that enables Atoh1 to sustain its own expression and that of downstream targets through cooperative enhancer activation. *Tox3* was first cloned and characterized as a calcium-dependent transactivator in *c-fos* expression in neurons (38). Its implication in neuron survival and subventricular zone neural stem cell function has also been suggested in cell culture and knockdown studies (39, 40). However, none of these investigations had supporting genetic evidence from mice lacking *Tox3*. In this study, we report that genetic inactivation of *Tox3* disrupts Atoh1 autoregulation, leading to failure of GNP maintenance despite intact initial lineage specification. This distinction is underscored by the differing phenotypes of *Atoh1*-null vs. *Tox3*- or *Atoh1* 3′ enhancer–deficient mice: whereas loss of Atoh1 abolishes GN formation entirely (11), loss of *Tox3* or *Atoh1* enhancer permits lineage establishment but compromises progenitor amplification. These findings support a modular view of transcriptional networks in which lineage identity and lineage expansion are governed by separable regulatory mechanisms.

The cooperative interaction between Tox3 and Atoh1 may exemplify a broader principle of context-dependent transcriptional regulation that extends beyond the cerebellum. In many developing tissues, lineage-defining factors such as Atoh1 act within regulatory environments shaped by cofactors that refine their activity. Tox3 could serve as such a modulator, integrating environmental or epigenetic cues to tailor Atoh1-driven gene programs to specific organ contexts. For instance, both *Tox3* (Fig. 1*F*) and Atoh1 are expressed in the embryonic spinal cord, where ATOH1 defines progenitors of the dorsal interneuron 1 population (41–44). Similarly, in the intestine, Atoh1 governs the differentiation of secretory lineages (45), while Tox3 has been implicated in enteroendocrine development—its loss in mouse intestinal organoids reduces serotonin-producing cells and increases ghrelin-producing cells (46). These observations raise the possibility that Tox3 may act as a conserved Atoh1 coregulator across multiple organ systems, reinforcing the idea that transcriptional versatility arises through modular cofactor engagement rather than wholesale rewiring of lineage determinants.

Beyond development, this network architecture has implications for disease states characterized by aberrant progenitor expansion. SHH-driven medulloblastoma arises from dysregulated GNP proliferation and accounts for ~30% of all medulloblastoma. Although pharmacologic inhibition of SHH signaling has shown clinical promise (47–51), its efficacy is limited by skeletal growth complications in young children and the emergence of resistance mutations in pathway components such as SMO (52). We show that deletion of *Tox3* prolongs survival in a SHH-driven mouse model, suggesting that Tox3 may represent a tractable, previously unrecognized therapeutic target in SHH-driven medulloblastoma. Strategies that disrupt the Tox3-Atoh1 interaction or inhibit Tox3 function could potentially complement or substitute for current SHH pathway inhibitors. In this context, Tox3 does not initiate tumorigenesis but sustains the transcriptional output required for tumor maintenance, highlighting the potential vulnerability of coregulatory nodes within oncogenic transcriptional networks.

Our findings also provide insights into the evolution of the vertebrate cerebellum (Fig. 8*D*). The cerebellum has undergone dramatic expansion across vertebrate lineages, with the development of the external granule layer and prolonged GNP proliferation thought to underlie increased cerebellar complexity, especially in mammals and birds (53). We show that the synergy between Tox3 and Atoh1 in activating a conserved E-box element within the Atoh1 3’ enhancer is conserved from fish to mammals. Moreover, single-cell RNA-seq analyses across species reveal that prolonged coexpression of *Tox3* and *Atoh1* correlates with an extended proliferative phase and larger cerebellar size. These results suggest that modulation of the Tox3-Atoh1 regulatory axis may have contributed to cerebellar expansion during vertebrate evolution. One limitation of our current analysis is the restricted availability of high-quality single-cell RNA-seq datasets from diverse species. Inclusion of taxa with unusually large and complex cerebella, such as electric fish (54), may further provide insights into the role of Tox3 in cerebellar evolution.

Finally, our study also establishes a transcriptional regulator atlas through an unbiased, organ-wise comparative transcriptomic analysis across developing mammalian organs. This atlas was designed not merely as a descriptive resource, but as a strategy to uncover transcriptional network logic by prioritizing regulators that are both tissue-enriched and developmentally dynamic. Compared with existing resources such as ENCODE and Tabula Muris, our analysis is optimized for transcription factor detection and highlights regulators with uncharacterized knockout phenotypes. Tabula Muris profiles adult (10 to 15 wk) mouse tissues only and therefore does not capture developmental stages, focusing instead on organ-level transcription factor correlations among shared cell types (55). In contrast, ENCODE primarily maps functional genomic elements–including coding, noncoding, and regulatory regions rather than tissue-specific transcription factor expression and perturbation profiles (56). The identification of *Tox3* through this approach illustrates the utility of this atlas for discovering coregulatory components that shape transcriptional network behavior during organogenesis.

In summary, this study advances understanding of transcriptional network logic by demonstrating how robust developmental gene expression can be achieved through cooperative interactions between lineage-defining transcription factors and context-specific coregulators. By positioning Tox3 as a transcriptional amplifier that stabilizes Atoh1-dependent programs, our findings provide a generalizable framework for how developmental, oncogenic, and evolutionary processes exploit modular network architecture to balance robustness with flexibility.

## Materials and Methods

Detailed methods are provided in *SI Appendix*, including reagents, animal models, histology, phylogenetic analysis, single-nucleus and bulk RNA-seq, ChIP-seq, RT-qPCR, coimmunoprecipitation, luciferase assays, and statistical analysis.

## Supplementary Material

Appendix 01 (PDF)

Dataset S01 (XLSX)

Dataset S02 (XLSX)

Movie S1. Global deletion of *Tox3* results in severe ataxia, related to Figure 2. Video recordings of postnatal day 31 mice are shown. At the start of the video, the larger mouse in the center is *Tox3+/+*, and the two smaller mice on either side are *Tox3−/−*.

Movie S2. Nervous system-specific deletion of *Tox3* leads to the onset of ataxia around postnatal day 10 (P10), related to Figure 2. Videos of P10 mice are shown. Left, *Tox3^f/f^*; right, *Nestin-Cre;Tox3^f/f^*.

Movie S3. Nervous system-specific deletion of *Tox3* causes severe ataxia by postnatal day 14 (P14), related to Figure 2. Videos of P14 mice are shown. Left, *Tox3^f/f^*; right, *Nestin-Cre;Tox3^f/f^*.

## Data Availability

Bulk RNA-seq, ChIP-seq, and snRNA-seq data generated from *Tox3* control and *Nestin-Cre;Tox3^f/f^* mutant mice in this study have been deposited in the Gene Expression Omnibus (GEO) under accession number GSE312658 (57). All other data are included in the manuscript and/or supporting information.
