# Supplementary material for: A transcription regulator atlas identifies TOX3 as an Atoh1 coactivator in cerebellar development and tumorigenesis
Source: Proc Natl Acad Sci U S A. 2026 Mar 18;123(12):e2527163123. doi: 10.1073/pnas.2527163123 (PMC13012119; doi:10.1073/pnas.2527163123)
Supplement: Supplementary file 1 — Appendix 01 (PDF) [file pnas.2527163123.sapp.pdf]

PNAS

# Supporting Information for

## A transcription regulator atlas identifies TOX3 as an Atoh1 co-activator in cerebellar development and tumorigenesis

Xiaoxin Chen[1,2,9], Xiaochen Zhong[1,2], William Yue[3], Bruce Wang[3], Brian Woo[4], Hani Goodarzi[4], Zaili Luo[5], Q. Richard Lu[6], Frédéric Flamant[7], Jeremy F. Reiter[4,8], Guo N. Huang[1,2,9]

[1]Cardiovascular Research Institute & Department of Physiology, University of California, San Francisco, San Francisco, CA, 94158, USA.

[2]Eli and Edythe Broad Center for Regeneration Medicine and Stem Cell Research, University of California, San Francisco, San Francisco, CA, 94158, USA.

[3]Department of Medicine and Division of Gastroenterology, University of California, San Francisco, San Francisco, CA, USA.

[4]Department of Biochemistry and Biophysics, University of California, San Francisco, San Francisco, CA, USA.

[5]Department of Pediatrics, University of Alabama at Birmingham, Birmingham, AL, United States

[6]Brain Tumor Center, Division of Experimental Hematology and Cancer Biology, Cincinnati Children's Hospital Medical Center, Cincinnati, OH, 45229, USA

[7]Ecole Normale Supérieure de Lyon, INRAE, CNRS, Institut de Génomique Fonctionnelle de Lyon, 69364 Lyon, France

[8]Chan Zuckerberg Biohub, San Francisco, CA 94158, USA.

[9]Corresponding authors. Xiaoxin Chen and Guo N. Huang

Email: Xiaoxin.Chen@ucsf.edu (X.C.) and Guo.Huang@ucsf.edu (G. N. H.)

**This PDF file includes:**

- Supplementary Materials and Methods
- Supplementary References
- Supplementary Figures S1 to S9
- Supplementary Table S1
- Legends for Movies S1 to S3
- Legends for Supplementary Dataset S1 and S2

**Other supporting materials for this manuscript include the following:**

- Movies S1 to S3
- Dataset S1 and S2

## Supplementary Materials and Methods

### Antibodies and reagents

Rabbit anti-TOX3 antibody (Sigma-Aldrich, Cat#HPA040376), rabbit anti-ATOH1 antibody (Proteintech, Cat#21215-1-AP), Ki-67 monoclonal antibody (SolA15) eFluor 570 (Thermo Fisher Scientific, Cat#41-5698-80), anti-phospho-Histone H3 (Ser10) antibody, clone 3H10 (Sigma-Aldrich, Cat# 05-806), adenylate cyclase 3 polyclonal antibody (Thermo Fisher Scientific, Cat#ab136648), anti-ARL3B antibody (Abcam, Cat#ab136648), rabbit anti-Calbindin D-28k antibody (Swant Swiss Antibodies, Cat#CB-38a), monoclonal anti-Flag(R) M2-peroxidase (HRP) antibody (Sigma-Aldrich, Cat#A8592), HA tag monoclonal antibody (Thermo Fisher Scientific, Cat# 26183-HRP), anti-rabbit IgG HRP-linked antibody (Cell Signaling, Cat#7074), normal rabbit IgG antibody (Cell Signaling, Cat#2729), wheat germ agglutinin (WGA) CF488A conjugate (Biotium, Cat#29022-1), DAPI-fluoromount-G clear mounting media (Southern Biotech, Cat#0100-20).

### Plasmids

The *Atoh1* E-box region sequences and their mutant variants were synthesized by IDT and cloned into the pGL3 TATA plasmid, where the 58-bp basal promoter of Hsp70 was inserted upstream of a firefly luciferase coding sequence (*1*). The CDS with HA/FLAG tag sequence of zebrafish *tox3* (NCBI Ref: XM_005169018.4), zebrafish *atoh1a* (NCBI Ref: NM_131091.2), frog *tox3* (NCBI Ref: NM_001128012.1), frog *atoh1* (NCBI Ref: XM_004911085.4), lizard *TOX3* (NCBI Ref: XM_035120017.2), and lizard *ATOH1* (NCBI Ref: XM_035112926.2) were synthesized by GeneScript and subcloned into pcDNA3.1-CMV-P2A-eGFP plasmids between the CMV and P2A site using KpnI and XbaI site for digestion and ligation. The ORF of human *TOX3* with an HA tag was PCR-amplified from pCMV-IRES-EGFP-TOX3 vector (*2*), and cloned into pAdTrack-CMV vector (Addgene # 16405). The ORF of mouse *Atoh1* (NCBI Ref: NM_007500.5) with a Flag tag were PCR-amplified from cDNA and cloned into pAdTrack-CMV vector. In pAdTrack-CMV vector, the transgene is under the control of a CMV promoter and a separate CMV promoter directs GFP expression. Truncated versions of *TOX3* were PCR-amplified from the human pAdTrack-TOX3 construct and subcloned into the pRK5-HA vector (3).

### Animals

Mice procedures were conducted in accordance with the Institutional Animal Care and Use Committee (IACUC) of the University of California, San Francisco under animal protocol AN18341. *Nestin-Cre* (strain B6.Cg-Tg(Nes-cre)1Kln/j, stock number: 003771, from The Jackson Laboratory) and *Tox3*$^{tm1a(KOMP)Mbp}$ (purchased from KOMP Repository, University of California, Davis) mouse lines were maintained according to the University of California, San Francisco institutional guidelines. For all experiments, both male and female animals were used, and no gender difference was observed.

**Generation of mouse lines**

Mice heterozygous for the *Tox3*$^{tm1a(KOMP)Mbp}$ transgene were crossed with mice carrying the FLP allele to obtain floxed (*Tox3*$^{f/+}$) allele, which was then crossed with mice heterozygous for *Nestin-Cre* transgene to generate mice with *Nestin-Cre;Tox3*$^{f/+}$ transgene. *Nestin-cre;Tox3*$^{f/+}$ mice were further crossed with *Tox3*$^{f/f}$ mice to obtain *Nestin-Cre;Tox3*$^{f/f}$ and Cre-negative control mice.

**X-gal staining**

Embryos and organs were collected at indicated stages and stained for β-galactosidase activity. For β-galactosidase staining, embryos or tissues were fixed on ice for 30 minutes in 3.7 % paraformaldehyde (PFA) dissolved in phosphate-buffered solution (PBS), washed thrice with PBS at room temperature (10 minutes each), and stained overnight at 4 °C in an x-gal staining PBS buffer containing 4 mM K4Fe(CN)6, 4 mM K3Fe(CN)6, 2 mM MgCl2 and 1 mg/mL X-gal.

**Histology staining**

The organs were harvested and fixed in 3.7% PFA before paraffin embedding. Paraffin sections of 5 µm were obtained and baked for 20 minutes at 52°C to deparaffinize. Slides were placed in xylene for 10 minutes, rehydrated in gradient ethanol, and finally distilled water. Tissue sections were then stained in hematoxylin solution for 3 minutes, washed with distilled water for 2 minutes, stained in eosin for 1.5 minutes, and washed with distilled water. To dehydrate the sections, slides were placed in 90% ethanol for 2 minutes, 95% ethanol for 2 minutes, 100% ethanol twice (2 minutes each), and Xylene for 10 minutes. Slides were air-dried and mounted with Dako Mounting Medium. Sections were imaged using Axio Scan.Z1.

**Immunohistochemistry staining**

Transcardial perfusion with 3.7% PFA was performed before harvesting the cerebella. The cerebella were further fixed in 3.7% PFA for 24 h, washed 3 times with PBS and then allowed to sink in 30% sucrose for 3 days before embedding in O.C.T. Compound (Tissue Tek, cat#4583) and snap frozen. Embedded samples were then sectioned with a Leica CM3050S to 7 μm thickness. Tissue sections were then permeabilized in 0.1% Triton X-100 in PBS (PBST), blocked in 5% normal donkey serum in PBST for 30 min at room temperature, and incubated with primary antibodies in PBST overnight at 4°C. Sections were then incubated in their corresponding secondary antibodies (Alex Fluor 488, 55, 647; Invitrogen, 1:750) for 2 hours at room temperature. Nuclei were visualized by DAPI staining. The sections were then imaged using the Axio Scan.Z1 or a Nikon Eclipse Ti microscope with Nikon DS-Qi2 camera.

**Phylogenetic analysis**

Orthologous genes and similar genes for phylogenetic analysis were obtained from an NCBI database search. Orthologous gene groups were calculated by NCBI's Eukaryotic Genome Annotation pipeline for the NCBI Gene dataset, utilizing a combination of protein sequence similarity and local synteny information. The 'similar genes' group comprised a set of eukaryotic genes from the NCBI Gene database, selected based on a combination of calculated ortholog and protein architecture similarity. The alignment and generation of the phylogenetic tree were performed using NCBI's constraint-based multiple alignment tool (COBALT). COBALT utilizes various strategies, such as the conserved domain database, protein motif database, and sequence similarity through RPS-BLAST, BLASTP, and PHI-BLAST. By extracting pairwise constraints from these sources, COBALT incorporates them into a progressive multiple alignment approach, enhancing the accuracy and reliability of the alignment process.

**Tissue collection and nuclei dissociation**

Cerebella were collected from P1 *Nestin-cre;Tox3$^{f/f}$* and Cre-negative control mice, snap-frozen in dry ice, and stored at -80°C for single-nuclei extraction. Single nuclei were generated from 12 snap-frozen cerebella from each condition using the Singulator™ 100 system (S2Genomics, Livermore, CA), following the regular nuclei isolation protocol. Nuclei preparations were cleaned as follows: nuclei were centrifuged at 500 g for 5 min, resuspended in 2 mL of cold Storage Buffer (S2Genomics), and filtered through a 40 μm Flowmi Tip Strainer filter. The filtered nuclei were then resuspended in 2 mL of Storage

Buffer, and centrifuged again at 500g for 5 min. After centrifugation, nuclei were resuspended in 200 µL of Storage Buffer supplemented with 1 U/µL of RNAse inhibitor (Sigma Aldrich, cat: 3335402001) and counted using a LUNA-FL™ Dual Fluorescence Cell Counter (Logo Biosystems, Anyang-si, South Korea).

**Single-nucleus RNA-seq library preparation and sequencing**

We pooled 12 control cerebella and 12 mutant cerebella from P1 animals to generate one control library and one mutant library. Single-nucleus RNA-seq libraries were generated using the Chromium Next GEM Single Cell 3' v.3.1 according to the manufacturer's protocol (10x Genomics), targeting 10,000 nuclei per sample. We performed 12 cycles for cDNA amplification for both samples. To generate the final dual indexed 10x libraries, 13 cycles were performed.

**Library pooling and quality control**

After library preparation, individual libraries were quality checked on an Agilent 4200 Tapestation using D1000 screen tape. The libraries were pooled equally molar at a final concentration of 5.7 nM and quality checked again on an Agilent 4200 Tapestation using a D1000 screen tape.

**Sequencing and alignment**

Individual pools of 10x 3' gene expression libraries were sequenced on Illumina's NovaSeq S4 flow cells, targeting a sequencing read depth of 100,000 reads per cell. Sequencing was performed by UCSF Center for Advanced Technology with the following sequencing parameters: Read 1= 28 cycles, Index 1= 10 cycles, Index 2= 10 cycles, Read 2= 90 cycles. The sequences were de-multiplexed using bcl2fastq. Reads were aligned to a Gencode Reference vM23/Ensembl 98 genome provided by 10X using CellRanger version 7.0.1, available from Computational Biology and informatics (CBI) at UCSF.

**snRNA-seq quality control**

Reads mapped to the mouse genome at rates of 87.6% and 88.7% for control and mutant cerebella libraries, respectively. Prior to quality-control filtering, 4,857 nuclei from control cerebella and 6,946 nuclei from mutant cerebella were recovered. Sequencing produced a mean of 189,767 and 117,030 reads per nucleus for control and mutant samples, respectively, with a median of 2,143 and 2,153 genes detected per nucleus. Quality filtering was performed using the Seurat R package (*4*). To remove ambient RNA, we

utilized SoupX (*5*). Scrublet was used to identify and remove doublets from downstream analysis (*6*). Cells with more than 25% of transcripts originating from mitochondrial genes were excluded. Additionally, genes expressed in fewer than 10 nuclei were removed. After quality filtering and removal of unwanted cells, the data were normalized based on total expression, multiplied by a scale factor of 10,000 and log transformed.

**Integrated analysis of snRNA-seq datasets**

To account for potential batch effects, we utilized the IntegrateData function implemented in Seurat V3, which employs canonical correlation analysis to identify a linear combination of features that construct a shared correlation structure and align the global transcriptome across datasets (*4*). We performed standard preprocessing (log-normalization) and identified the top 3000 variable features for each individual dataset. Integration anchors were identified using the FindIntegrationAnchors function with default parameters and a dimension of 30. These anchors were then passed to the IntegrateData function to generate an integrated Seurat object.

**Visualization and clustering**

For data visualization, we employed Uniform Manifold Approximation and Projection (UMAP) to project cells in a 2D space based on the aligned canonical correlation analysis (*4*). Aligned canonical correlation vectors (1:20) were used to identify clusters using a shared nearest neighbor modularity optimization algorithm. We applied graph-based clustering, dividing cells into 23 clusters using the FindCluster function in Seurat with resolution 0.8. Granule neuron progenitors and granule cell clusters were identified based on the expression of *Gli2* and *Pax6,* while Purkinje cell clusters were identified based on *Calb1* expression. One cluster containing cell doublets and low-quality cells was removed.

**Bulk RNA-sequencing and analysis**

Total RNA was extracted from P1 cerebella of the indicated genotypes using TRIzol$^{TM}$ reagent (Invitrogen, #15596026) following the manufacturer's instructions. RNA-seq was performed by Novogene Inc. The procedure involved mRNA purification using poly-T oligo-attached magnetic beads, fragmentation, first and second strand cDNA synthesis, end repair, A-tailing, adapter ligation, size selection, amplification, and purification to generate the library. Library quantification and size distribution detection were performed

using Qubit, real-time PCR, and a bioanalyzer. The quantified libraries were pooled and sequenced on Illumina platforms based on effective library concentration and data requirements. Sequencing generated 150 bp paired-end reads, which were processed by removing reads containing adapters, ploy-N, and low-quality reads. The clean reads were aligned to the mm10 reference genome using Hisat2 v2.0.5 (*7*). FeatureCounts v1.5.0 (*8*) was used to count the number of reads mapped to each gene, and FPKM values were calculated based on gene length and read count. Differential expression analysis between two conditions/groups was performed using the DESeq2 R package v1.20.0 (*9*). The resulting *P*-values were adjusted using the Benjamini and Hochberg's approach to control the false discovery rate. A threshold of P-adj <0.01 and |Log2 FC| > 0.5 was used to identify significant up- and down-regulated genes.

**ChIP-sequencing and analysis**

Chromatin from the cerebellum was prepared from P1 *Nestin-cre;Tox3$^{f/f}$* and *Tox3$^{f//f}$* or *Tox3$^{f/+}$* cerebella. Frozen cerebella were dissociated with a 1 mL pipette until no large tissue chunks were visible and then fixed in 1% formaldehyde in PBS for 20 min at room temperature. The fixation was stopped by adding glycine to a final concentration of 125 mM for 5 min at room temperature. The samples were incubated with cell lysis buffer (50 mM Tris-Cl, pH 8.0, 140 mM NaCl, 1 mM EDTA, 10% glycerol, 0.5% NonidetP-40 substitute (Fluka #74385), 0.25% Triton X-100, 1:200 protease inhibitor cocktail (Sigma-Aldrich #P8340)) and homogenized using a glass Dounce homogenizer with a type "B" pestle, performing 15 strokes. After centrifugation, the nuclear pellet was resuspended in nuclear lysis buffer (10mM Tris-Cl, pH 8.0, 1 mM EDTA, 0.5 mM EGTA, 0.2% SDS, 1:200 protease inhibitor cocktail) and incubated for 20 min on ice. Samples were sonicated for 12 cycles of 20s on and 60s off using Branson Digital Sonifier model 450 at 9% amplitude to generate fragments of 200-500 bp. ChIP was performed using 2 µg of anti-TOX3 antibody (Sigma). Following ChIP, ChIP-seq libraries were generated using the NEBNext® Ultra™ II DNA Library Prep Kit and sequenced using 150 bp paired-end reads on an Illumina Hiseq X platform. For analysis, adapter sequences were removed from the raw reads using Trimgalore v0.6.7. Reads were aligned to the primary assembly of the mm10 mouse genome using Bowtie v2.5.0 with default parameters (*10*). Duplicates were removed using Sambamba v1.0 (*11*). Blacklisted regions identified by the ENCODE project were filtered out from the final BAM files. Signal files were generated with deeptools

bamCoverage v3.5.1 (*12*). Binding regions were identified by comparing pooled Tox3 ChIP-seq signal with input signal, with a q value of 5.00E-02 using the callpeak function in MACS2 v2.2.7.1 (*13*). All MACS2 peaks were annotated with TSS of the nearest gene using HOMER v4.11 (*14*). Data were visualized using IGV v2.11.2 (*15*). Gene ontology analysis of the top 3,000 peak-related genes was performed using Metascape (*16*).

**RT-qPCR analysis**

Total RNA was isolated using TRIzol, and cDNA was synthesized using iScript™ cDNA Synthesis Kit. qPCR was performed using the SYBR Select Master Mix (Applied Biosystems, 4472908) and the QuantStudio 5 Real-Time PCR system (Applied Biosystems). Fold changes in transcript levels were determined using the ΔΔCt method. Primers for *Tox3*, *Ccna1*, *Ccnb1*, *Ccnb2*, *Cdca8*, *Cdk1*, *Pcna*, *Mki67*, and *Actb* are listed below.

| Primer name | Sequence |
|---|---|
| *Ccna1* forward | 5' - TGATGCTTGTCAAATGCTCAGC - 3' |
| *Ccna1* reverse | 5' - AGGTCCTCCTGTACTGCTCAT - 3' |
| *Ccnb1* forward | 5' - AAGGTGCCTGTGTGTGAACC - 3' |
| *Ccnb1* reverse | 5' - GTCAGCCCCATCATCTGCG - 3' |
| *Ccnb2* forward | 5' - GCCAAGAGCCATGTGACTATC - 3' |
| *Ccnb2* reverse | 5' - CAGAGCTGGTACTTTGGTGTTC - 3' |
| *Cdca8* forward | 5' - CAAATTGAGTCCGACAGACAGA - 3' |
| *Cdca8* reverse | 5' - GCCGAAGGATCTCGATGTTGT - 3' |
| *Cdk1* forward | 5' - AGAAGGTACTTACGGTGTGGT - 3' |
| *Cdk1* reverse | 5' - GAGAGATTTCCCGAATTGCAGT - 3' |
| *Pcna* forward | 5' - TTTGAGGCACGCCTGATCC - 3' |
| *Pcna* reverse | 5' - GGAGACGTGAGACGAGTCCAT - 3' |
| *Mki67* forward | 5' - ATCATTGACCGCTCCTTTAGGT - 3' |
| *Mki67* reverse | 5' - GCTCGCCTTGATGGTTCCT - 3' |
| *Actb* forward | 5' - AGTGTGACGTTGACATCCGT - 3' |
| *Actb* reverse | 5' - TGCTAGGAGCCAGAGCAGTA - 3' |
| *Tox3* forward | 5' - CCTTGGGGAGGAGCAAAAGCA - 3' |
| *Tox3* reverse | 5' - AGCAGCAGCCTTGGAAACGAG - 3' |

**Co-immunoprecipitation assays**

For co-immunoprecipitation analysis using cerebellar tissue, three to five P7 mouse cerebella were collected and lysed in 1% PBST containing 1:200 protease inhibitor

cocktail followed by sonication at 20 % amplitude for 20 sec, repeated twice. Afterwards, the lysate was spun at 15,000 rpm for 10 min, twice. The supernatant was mixed with 1 μg anti-TOX3 antibody (Sigma) overnight at 4°C. Then, the mixture was incubated with Dynabeads for 2 hrs. The immunoprecipitated complex was washed with TBST three times before elution by SDS loading buffer. For co-immunoprecipitation analysis using overexpressed proteins, the specified HA and FLAG fusion proteins were expressed in HEK293 cells. Cells were lysed in 1% PBST containing 1:200 protease inhibitor cocktail. After sonication, spin at 13,000 rpm for 10 min. The supernatant was mixed with 1 μg anti-FLAG M2 antibody (Sigma) overnight at 4°C. The mixture was incubated with Dynabeads for additional 2 to 3 hrs. The immunoprecipitated complex was washed with TBST three times before elution by SDS loading buffer.

**Luciferase assay**

High-purity plasmids were prepared using QIAprep 2.0 spin columns (Qiagen). HEK293T cells were transiently transfected in a 48-well plate at 70–80% confluence with 18.75 ng of the indicated pGL3-Basic-TATA-E-box-firefly luciferase reporter plasmid, 0.375 ng of a SV40-Renilla control plasmid (*1*), and 37.5 ng of the indicated expression constructs using polyethyleneimine (Sigma). The cells were then washed and lysed in 100 μl of passive lysis buffer 48 h after transfection (Promega), and 20 μl of the lysate was transferred to a white 96-well microtiter plate. A total of 100 μl of luciferase assay buffer (Promega) was directly injected into each well prior to measurement. Luminescence was measured using a microplate reader and integrated over 10 seconds. Normalization was conducted using dual luciferase assays, where 20 μl of lysate was transferred to a second white microtiter plate. Then, 100 μl of coelenterazine (Research Products International) in PBS buffer was injected into each well, and Renilla luminescence was measured as described above.

**Statistical analysis**

Error bars represent the standard error of the mean. The Mann-Whitney U test was used to compare difference between two groups, specifically Figure 2F and 3A. The Kruskal-Wallis test followed by Mann-Whitney U test was used for comparison in Figure 5A and 5C; Dunn's multiple comparisons were used for comparison among more than two groups in Figure 7B, 7D, and 8B. Lifespan analysis was performed by Kaplan–Meier survival analysis. All data are presented as mean ± standard error of the mean (SEM). A value of

*P*<0.05 was considered significant. All statistical analyses were performed using GraphPad Prism, version 9.0, software.

## Supplementary References


1. G. N. Huang, *et al.*, C/EBP transcription factors mediate epicardial activation during heart development and injury. *Science* **338**, 1599–1603 (2012).
2. A. Seksenyan, *et al.*, TOX3 is expressed in mammary ER(+) epithelial cells and regulates ER target genes in luminal breast cancer. *BMC Cancer* **15**, 22 (2015).
3. G. N. Huang, *et al.*, NFAT binding and regulation of T cell activation by the cytoplasmic scaffolding Homer proteins. *Science* **319**, 476–481 (2008).
4. A. Butler, P. Hoffman, P. Smibert, E. Papalexi, R. Satija, Integrating single-cell transcriptomic data across different conditions, technologies, and species. *Nat Biotechnol* **36**, 411–420 (2018).
5. M. D. Young, S. Behjati, SoupX removes ambient RNA contamination from droplet-based single-cell RNA sequencing data. *Gigascience* **9**, giaa151 (2020).
6. S. L. Wolock, R. Lopez, A. M. Klein, Scrublet: computational identification of cell doublets in single-cell transcriptomic data. *Cell Syst* **8**, 281-291.e9 (2019).
7. D. Kim, B. Langmead, S. L. Salzberg, HISAT: a fast spliced aligner with low memory requirements. *Nat Methods* **12**, 357–360 (2015).
8. Y. Liao, G. K. Smyth, W. Shi, featureCounts: an efficient general purpose program for assigning sequence reads to genomic features. *Bioinformatics* **30**, 923–930 (2014).
9. M. I. Love, W. Huber, S. Anders, Moderated estimation of fold change and dispersion for RNA-seq data with DESeq2. *Genome Biol* **15**, 550 (2014).
10. B. Langmead, S. L. Salzberg, Fast gapped-read alignment with Bowtie 2. *Nat Methods* **9**, 357–359 (2012).
11. A. Tarasov, A. J. Vilella, E. Cuppen, I. J. Nijman, P. Prins, Sambamba: fast processing of NGS alignment formats. *Bioinformatics* **31**, 2032–2034 (2015).
12. F. Ramírez, *et al.*, deepTools2: a next generation web server for deep-sequencing data analysis. *Nucleic Acids Res* **44**, W160-165 (2016).
13. Y. Zhang, *et al.*, Model-based analysis of ChIP-Seq (MACS). *Genome Biol* **9**, R137 (2008).
14. S. Heinz, et al., Simple combinations of lineage-determining transcription factors prime cis-regulatory elements required for macrophage and B cell identities. Molecular Cell **38**, 576–589 (2010).
15. J. T. Robinson, *et al.*, Integrative genomics viewer. *Nat Biotechnol* **29**, 24–26 (2011).
16. Y. Zhou, et al., Metascape provides a biologist-oriented resource for the analysis of systems-level datasets. Nat Commun 10, 1523 (2019).

# Supplementary Figures

**A**

| Organ (# of genes) | Gene Ontology | P-adjust |
|---|---|---|
| Brain (366) | Head development | 7.5858E-30 |
| | Central nervous system neuron differentiation | 2.3442E-17 |
| | Regulation of neuron differentiation | 9.3325E-13 |
| Cerebellum (272) | Brain development | 4.7863E-28 |
| | Hindbrain development | 2.6915E-15 |
| | Regulation of neuron differentiation | 3.2359E-13 |
| Heart (204) | Muscle structure development | 2.6303E-14 |
| | Cardiac chamber formation | 4.2658E-07 |
| | Muscle organ development | 4.7863E-07 |
| Kidney (208) | Embryonic morphogenesis | 5.4954E-44 |
| | Gland development | 1.8197E-18 |
| | Renal system development | 2.1878E-18 |
| Liver (313) | RNA biosynthetic process | 1.2589E-35 |
| | Chromatin organization | 8.3176E-25 |
| | Regulation of hemopoiesis | 1.4791E-10 |
| Ovary (190) | Regionalization | 3.6308E-15 |
| | In utero embryonic development | 7.2444E-06 |
| | Genitalia development | 9.3325E-05 |
| Testis (353) | Formation of primary germ layer | 1.9953E-07 |
| | Male gamete generation | 7.7625E-06 |
| | Male sex determination | 1.9055E-05 |

**B**

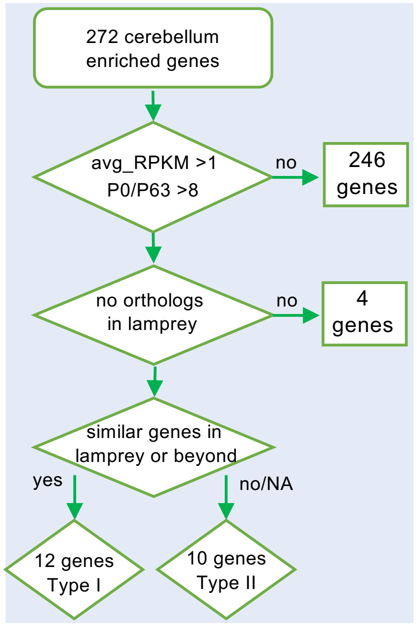


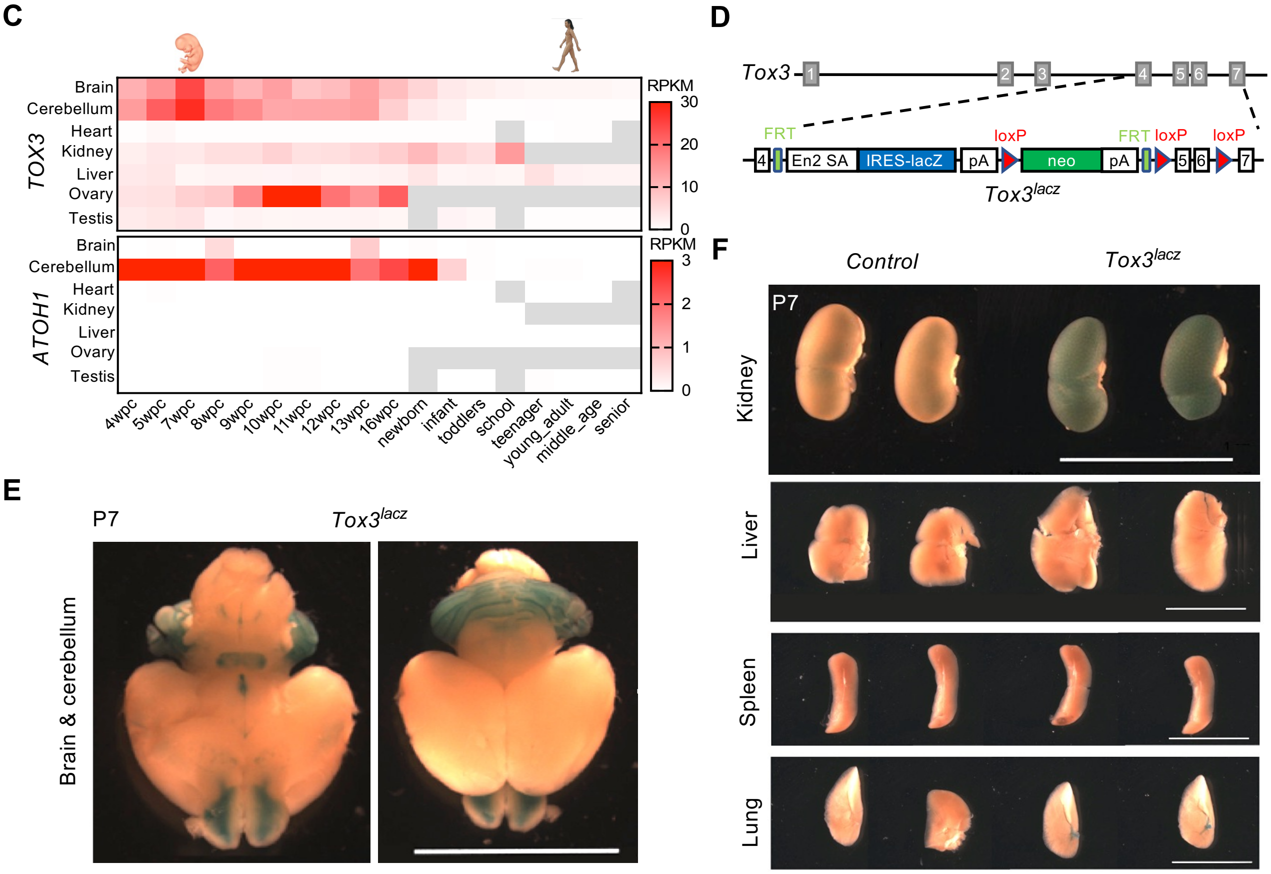

**Figure S1. Gene ontology, filtering strategy, and gene expression analysis.**
(A) Top GO terms associated with each group of tissue-enriched transcription factors. (B) Workflow for identifying candidate transcription factors involved in cerebellum expansion. (C) Heatmap showing the expression of *TOX3 and ATOH1* in major human organs at different developmental stages. Grey indicates unavailable data. Stages: newborn (0–20 days), infant (6–12 months), toddler (2–4 years), school (7–8 years), teenager (13–17 years), young adult (25–39 years), middle age (50–54 years), senior (58 year). Wpc, weeks post conception. (D) Schematic of the mouse *Tox3* locus and design of the transgenic construct for *LacZ* knockin and *Tox3* gene knockout. (E) X-gal staining of mouse cerebrum and cerebellum at postnatal day 7 (P7). (F) Representative X-gal staining of mouse kidneys, livers, spleens, and lungs at P7. Scale bars: 1 cm.

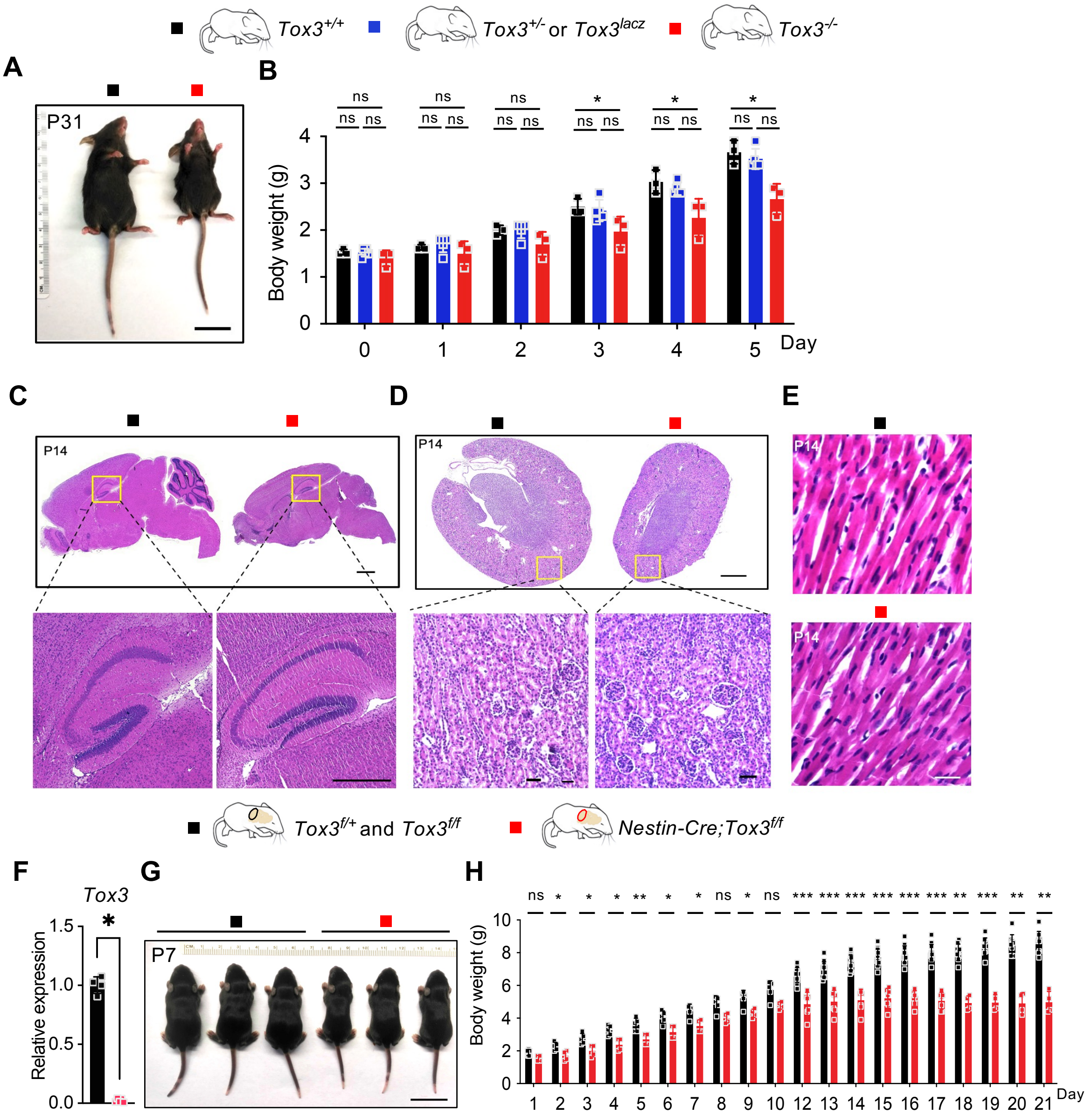


**Figure S2. Body weight and histological analyses of *Tox3* knockout mice.**
(A) Photos of control and knockout mice at postnatal day 31 (P31). (B) Body weight of neonatal transgenic mice ($Tox3^{+/+}$, n=3; $Tox3^{+/-}$, n=4; $Tox3^{-/-}$, n=3). (C) Representative histological images of brain sections from control and knockout mice at P14. Magnified views of the hippocampus are shown below (n=3 per genotype). (D) Representative images of kidney sections from control and knockout mice at P14 (n=3 per genotype). (E) Representative images of heart sections from control and knockout mice at P14 (n=3 per genotype). (F) Efficient deletion of *Tox3* in neural-specific knockout mice. Expression was measured by quantitative RT-PCR at P1 (control: $Tox3^{f/+}$ and $Tox3^{f/f}$, n=4 mice; *Nestin-Cre;*$Tox3^{f/f}$, n=5 mice). (G) Representative photos of control and mutant mice at P7. (H) Body weight changes of control and mutant mice during development (n=5–9 per genotype). Values are reported as mean ± SEM. ns, not significant; **P*<0.05, ***P*<0.01, ****P*<0.001. Scale bars: 2 cm (A), 1 mm (C), 500 μm (C insert), 500 μm (D), 50 μm (D insert), 20 μm (E), 1 cm (G).

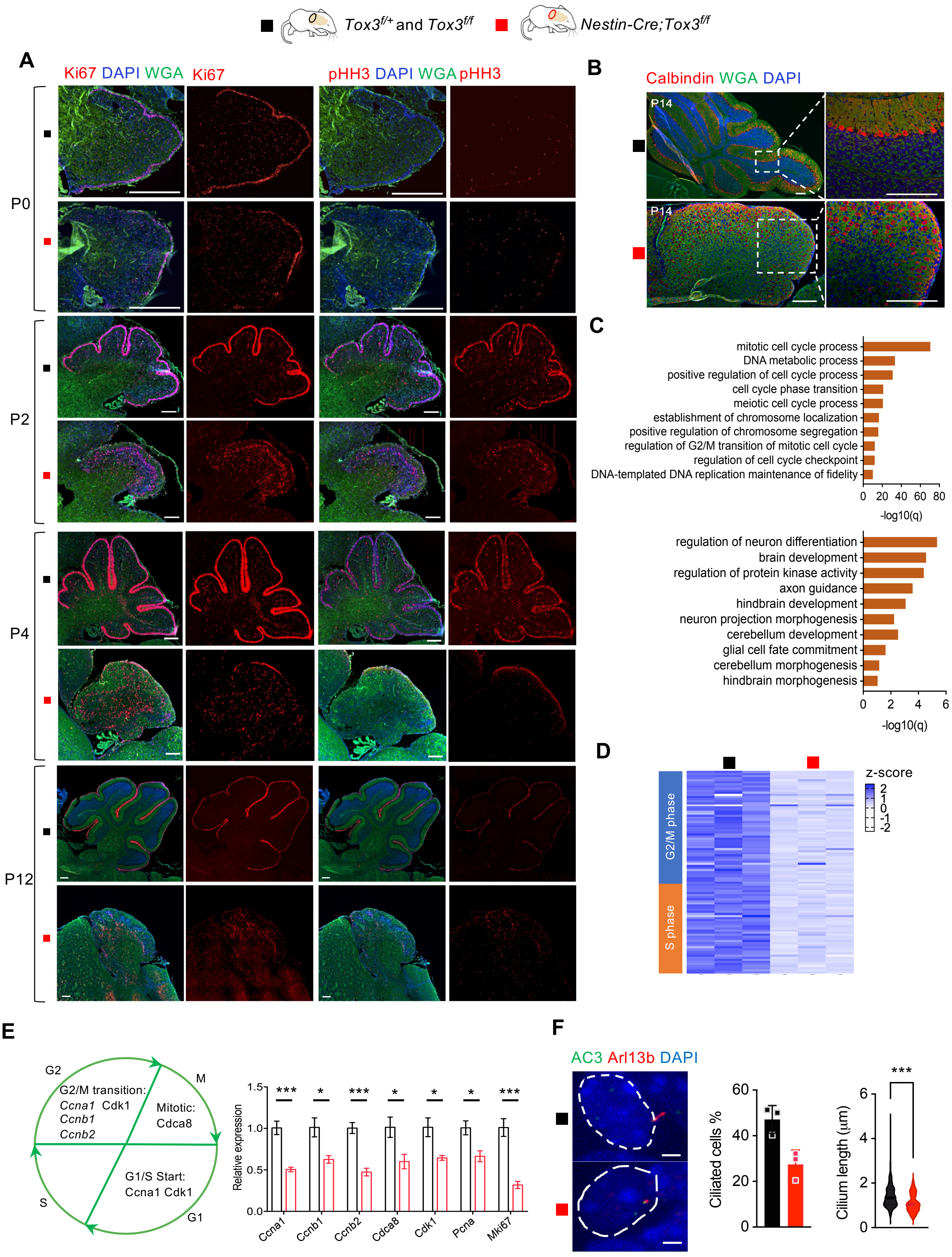

Tox3f/+ and Tox3f/f
Nestin-Cre;Tox3f/f
A
Ki67 DAPI WGA
Ki67
pHH3 DAPI WGA
pHH3
P0
P2
P4
P12
B
Calbindin WGA DAPI
P14
P14
C
mitotic cell cycle process
DNA metabolic process
positive regulation of cell cycle process
cell cycle phase transition
meiotic cell cycle process
establishment of chromosome localization
positive regulation of chromosome segregation
regulation of G2/M transition of mitotic cell cycle
regulation of cell cycle checkpoint
DNA-templated DNA replication maintenance of fidelity
-log10(q)
regulation of neuron differentiation
brain development
regulation of protein kinase activity
axon guidance
hindbrain development
neuron projection morphogenesis
cerebellum development
glial cell fate commitment
cerebellum morphogenesis
hindbrain morphogenesis
-log10(q)
D
z-score
G2/M phase
S phase
E
G2
M
S
G1
G2/M transition:
Ccna1 Cdk1
Ccnb1
Ccnb2
Mitotic:
Cdca8
G1/S Start:
Ccna1 Cdk1
Relative expression
Ccna1
Ccnb1
Ccnb2
Cdca8
Cdk1
Pcna
Mki67
F
AC3 Arl13b DAPI
Ciliated cells %
Cilium length (µm)

**Figure S3. Nervous system-specific deletion of *Tox3* impairs granule neuron progenitor proliferation in neonatal mice.**

(A) Representative images of Ki67 and phospho-histone H3 (pHH3) staining in control and mutant cerebella at postnatal day 0 (P0), P2, and P12. n=2–3 mice per genotype at each age. (B) Representative images of calbindin-stained cerebella from control and mutant mice at P14 (n=2–3 per genotype). (C) Bar plots showing the top 10 enriched Gene Ontology (GO) biological processes (top) and the top 10 non-cell cycle-related GO biological processes (bottom) from over-representation analysis of downregulated genes in mutant cerebella. (D) Heatmap showing the expression of 97 cell cycle-promoting genes in control and mutant samples. (E) Diagram illustrating the role of selected cell cycle genes in different phases of cell cycle progression with RT-qPCR validation of selected cell cycle-related genes (n=3 per genotype). (F) T*ox3* deficiency reduces both the proportion of ciliated cells and cilia length in cultured primary granule neuron progenitor cells. Representative images of AC3- and Arl13b-double-stained P1 granule neurons isolated from control and mutant cerebella. Dashed lines indicate nuclear boundaries (n=3 per genotype; 140 cells from *Nestin-Cre*;*Tox3*$^{f/f}$ mice and 61 cells from control *Tox3*$^{f/f}$ mice were analyzed). Values are reported as mean ± SEM. *$P$<0.05, ***$P$<0.001. Scale bars: 500 μm (A), 100 μm (B), 2 μm (F).

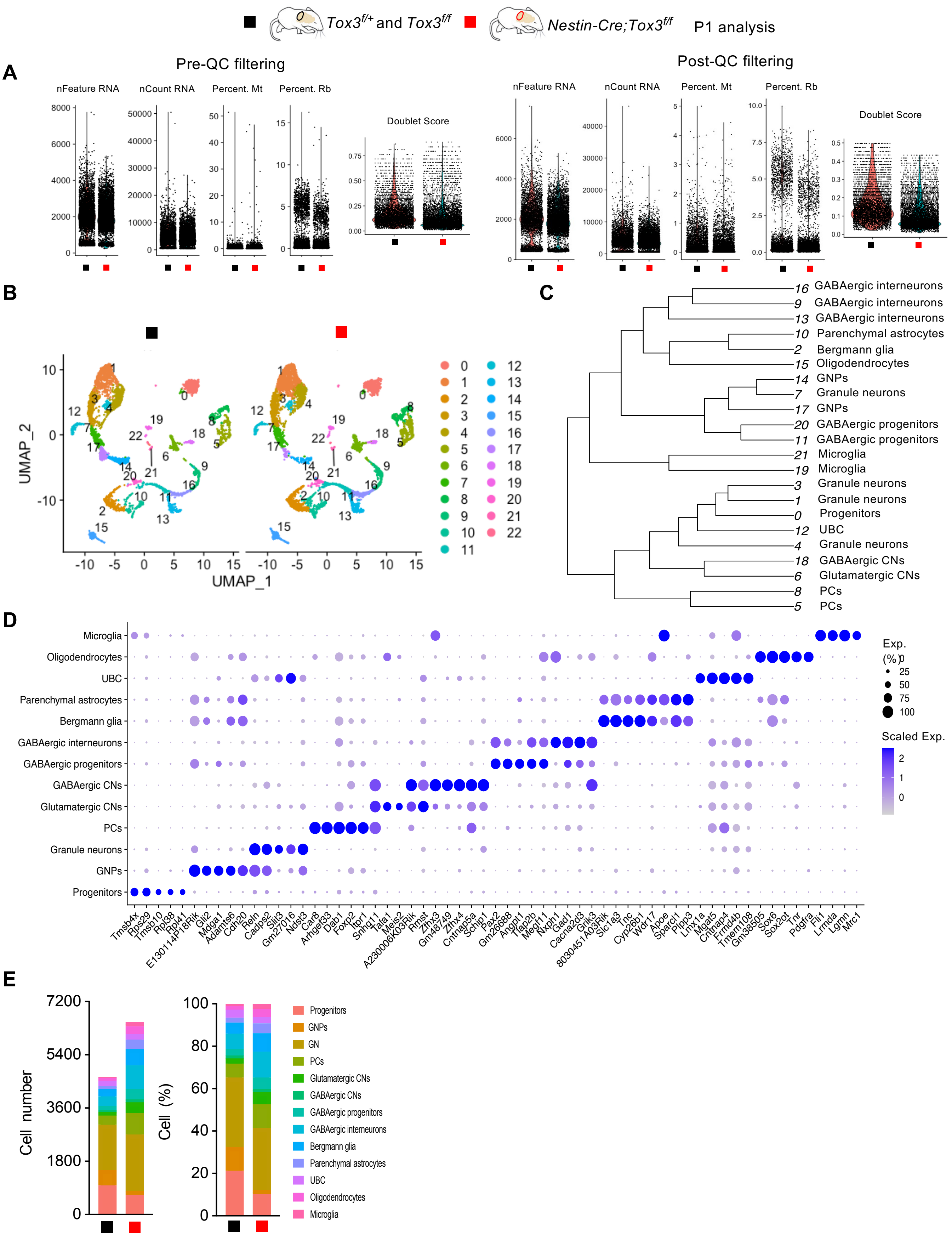

Tox3f/+ and Tox3f/f
Nestin-Cre;Tox3f/f
P1 analysis
A
Pre-QC filtering
nFeature RNA
nCount RNA
Percent. Mt
Percent. Rb
Doublet Score
Post-QC filtering
nFeature RNA
nCount RNA
Percent. Mt
Percent. Rb
Doublet Score
B
UMAP_2
UMAP_1
C
16 GABAergic interneurons
9 GABAergic interneurons
13 GABAergic interneurons
10 Parenchymal astrocytes
2 Bergmann glia
15 Oligodendrocytes
14 GNPs
7 Granule neurons
17 GNPs
20 GABAergic progenitors
11 GABAergic progenitors
21 Microglia
19 Microglia
3 Granule neurons
1 Granule neurons
0 Progenitors
12 UBC
4 Granule neurons
18 GABAergic CNs
6 Glutamatergic CNs
8 PCs
5 PCs
D
Microglia
Oligodendrocytes
UBC
Parenchymal astrocytes
Bergmann glia
GABAergic interneurons
GABAergic progenitors
GABAergic CNs
Glutamatergic CNs
PCs
Granule neurons
GNPs
Progenitors
Exp. (%)
25
50
75
100
Scaled Exp.
Tmsb4x
Rps29
Tmsb10
Rpl38
Rpl41
E130114P18Rik
Gli2
Mdga1
Adamts6
Cdh20
Reln
Cadps2
Slit3
Gm27016
Ndst3
Car8
Arhgef33
Dab1
Foxp2
Itpr1
Snhg11
Tafa1
Meis2
A230006K03Rik
Rmst
Zfhx3
Gm48749
Zfhx4
Cntnap5a
Schip1
Pax2
Gm26688
Angpt1
Tfap2b
Megf11
Nxph1
Gad1
Cacna2d3
Grik3
8030451A03Rik
Slc1a3
Tnc
Cyp26b1
Wdr17
Apoe
Sparcl1
Pipp3
Lmx1a
Mgat5
Cntnap4
Frmd4b
Tmem108
Gm38505
Sox6
Sox2ot
Tnr
Pdgfra
Fli1
Lrmda
Lgmn
Mrc1
E
Cell number
Cell (%)
Progenitors
GNPs
GN
PCs
Glutamatergic CNs
GABAergic CNs
GABAergic progenitors
GABAergic interneurons
Bergmann glia
Parenchymal astrocytes
UBC
Oligodendrocytes
Microglia

**Figure S4. Single-nucleus RNA-seq (snRNA-seq) identifies major cell types in the mouse cerebellum.**

(A) Violin plots showing the number of detected genes (nFeatur_RNA) and UMI counts (nCount_RNA), percentage of mitochondrial genes (percent.mt), percentage of ribosomal genes (percent.rb), and doublet scores before and after quality-control filtering for each group. (B) UMAP visualization of 23 clusters identified from the integrated dataset combining all samples, colored by cluster. Cluster22, which comprised broken cells, was excluded from subsequent analyses. (C) Cluster tree depicting the relationships and hierarchical classification among cell clusters. (D) Dot plot showing the expression of top marker genes for each cell cluster. (E) Bar graph showing the cell number and percentage of each cell type in each snRNA-seq sample.

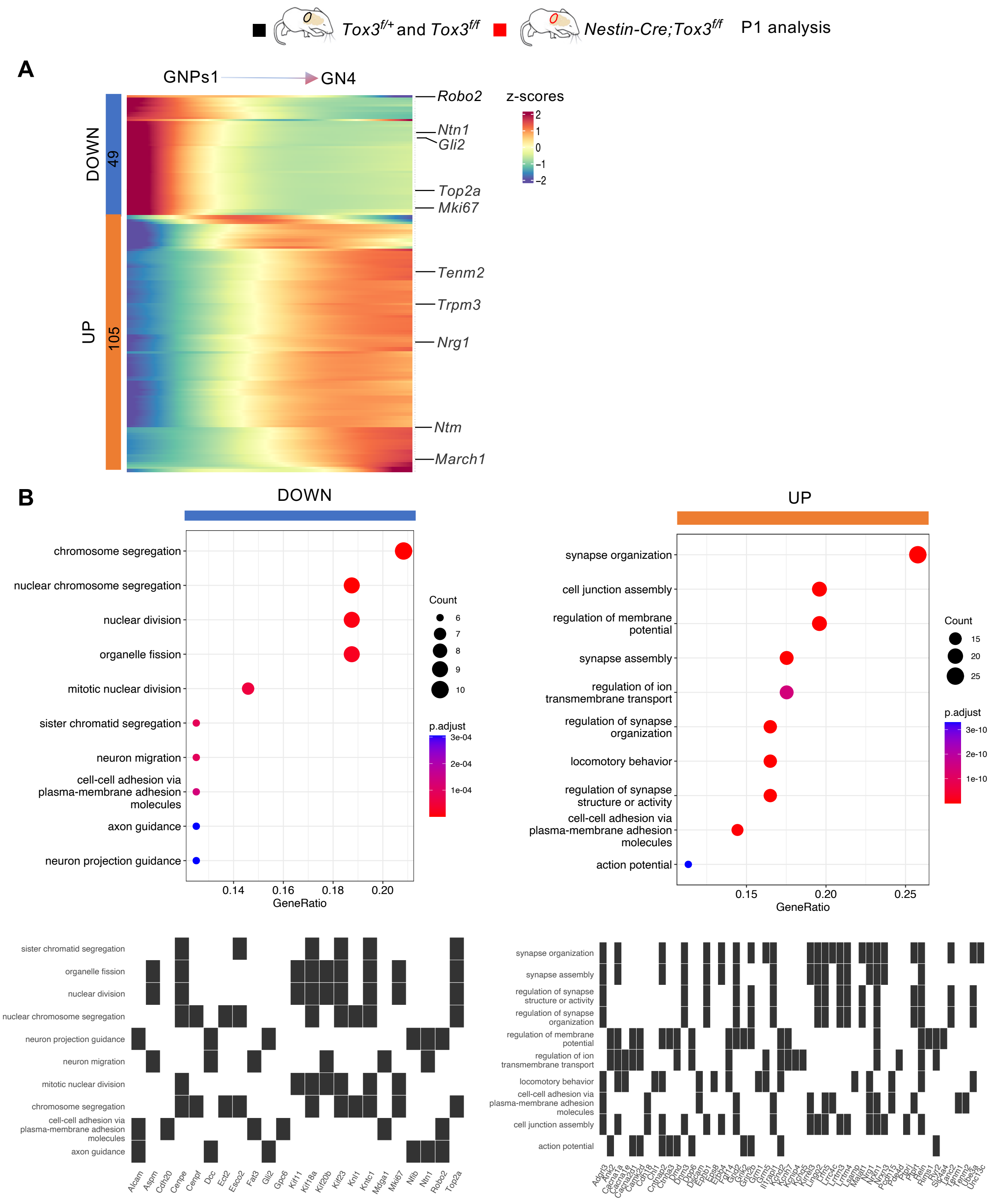


**Figure S5. Pseudotime trajectory analysis of granule neuron progenitor differentiation.**
(A) Heatmap depicting the differentiation trajectory from GNPs1 to GN4. (B) Dot plot and heatmap showing Gene Ontology (GO) term enrichment for differentially expressed genes identified along the pseudotime trajectory.

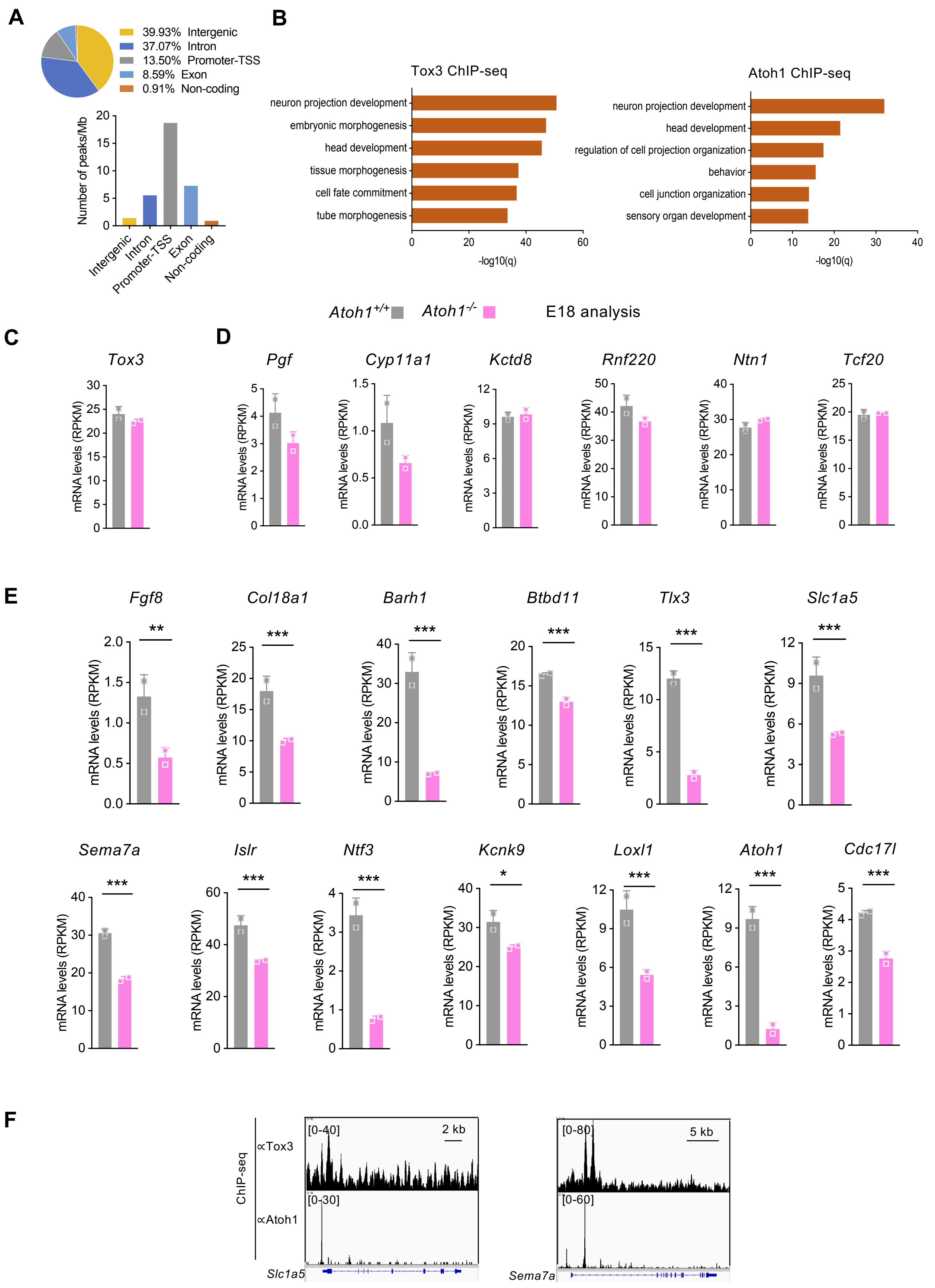
A
39.93% Intergenic
37.07% Intron
13.50% Promoter-TSS
8.59% Exon
0.91% Non-coding
Number of peaks/Mb
Intergenic
Intron
Promoter-TSS
Exon
Non-coding
B
Tox3 ChIP-seq
neuron projection development
embryonic morphogenesis
head development
tissue morphogenesis
cell fate commitment
tube morphogenesis
-log10(q)
Atoh1 ChIP-seq
neuron projection development
head development
regulation of cell projection organization
behavior
cell junction organization
sensory organ development
-log10(q)
Atoh1+/+ Atoh1-/- E18 analysis
C
Tox3
D
Pgf
Cyp11a1
Kctd8
Rnf220
Ntn1
Tcf20
mRNA levels (RPKM)
E
Fgf8
Col18a1
Barh1
Btbd11
Tlx3
Slc1a5
Sema7a
Islr
Ntf3
Kcnk9
Loxl1
Atoh1
Cdc17l
F
ChIP-seq
∝Tox3
∝Atoh1
[0-40]
2 kb
[0-30]
Slc1a5
[0-80]
5 kb
[0-60]
Sema7a

G

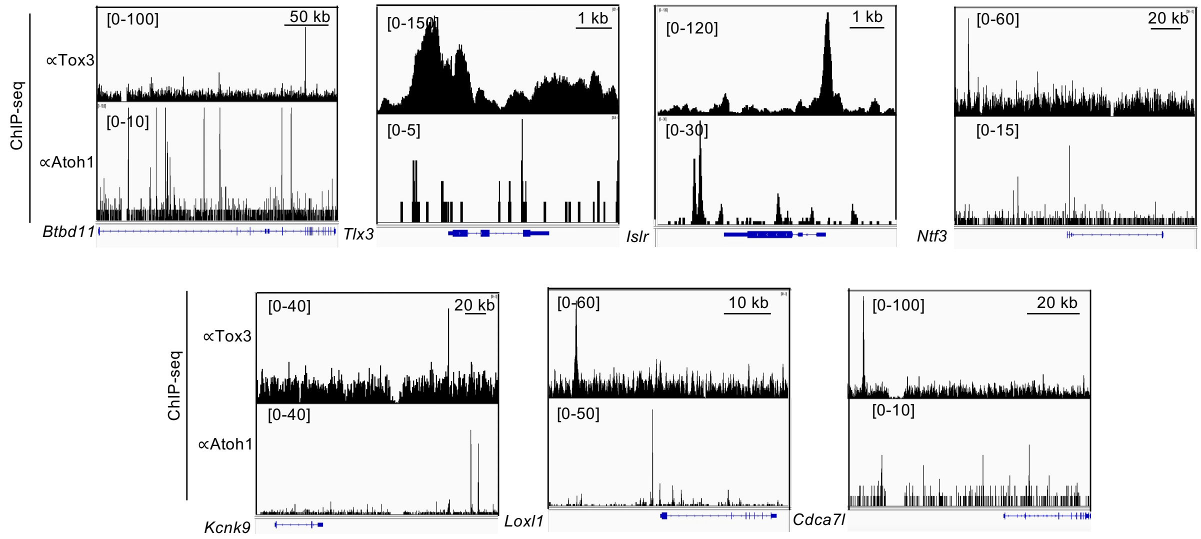


**Figure S6. Tox3 and Atoh1 ChIP-seq together with Atoh1 knockout gene expression analyses.**

(A) Genomic distribution and density of Tox3 ChIP-seq binding peaks. Mb, megabase. (B) Top six enriched Gene Ontology terms for genes associated with the top 3,000 Tox3 or Atoh1 binding sites. (C) RNA-seq analysis of embryonic day 18.5 (E18.5) cerebella from *Atoh1* knockout mice showing comparable *Tox3* expression between control and mutant samples. (D) RNA-seq analysis showing that, among the top 19 granule neuron progenitor-enriched genes shown in Figure 6E, six genes exhibit comparable expression levels in E18.5 *Atoh1* knockout cerebella (E) RNA-seq analysis showing that the remaining thirteen of the top 19 granule neuron progenitor-enriched genes are downregulated in E18.5 *Atoh1* knockout cerebella. (F) ChIP-seq analysis demonstrating overlapping Tox3 and Atoh1 binding peaks at the loci of two genes downregulated in *Atoh1* mutant mice, in addition to *Atoh1*, *Fgf8*, *Col18a1*, *Barh1* (shown in Figure 7A and C). (G) ChIP-seq analysis showing non-overlapping Tox3 and Atoh1 binding peaks at the loci of seven genes downregulated in *Atoh1* mutant mice.

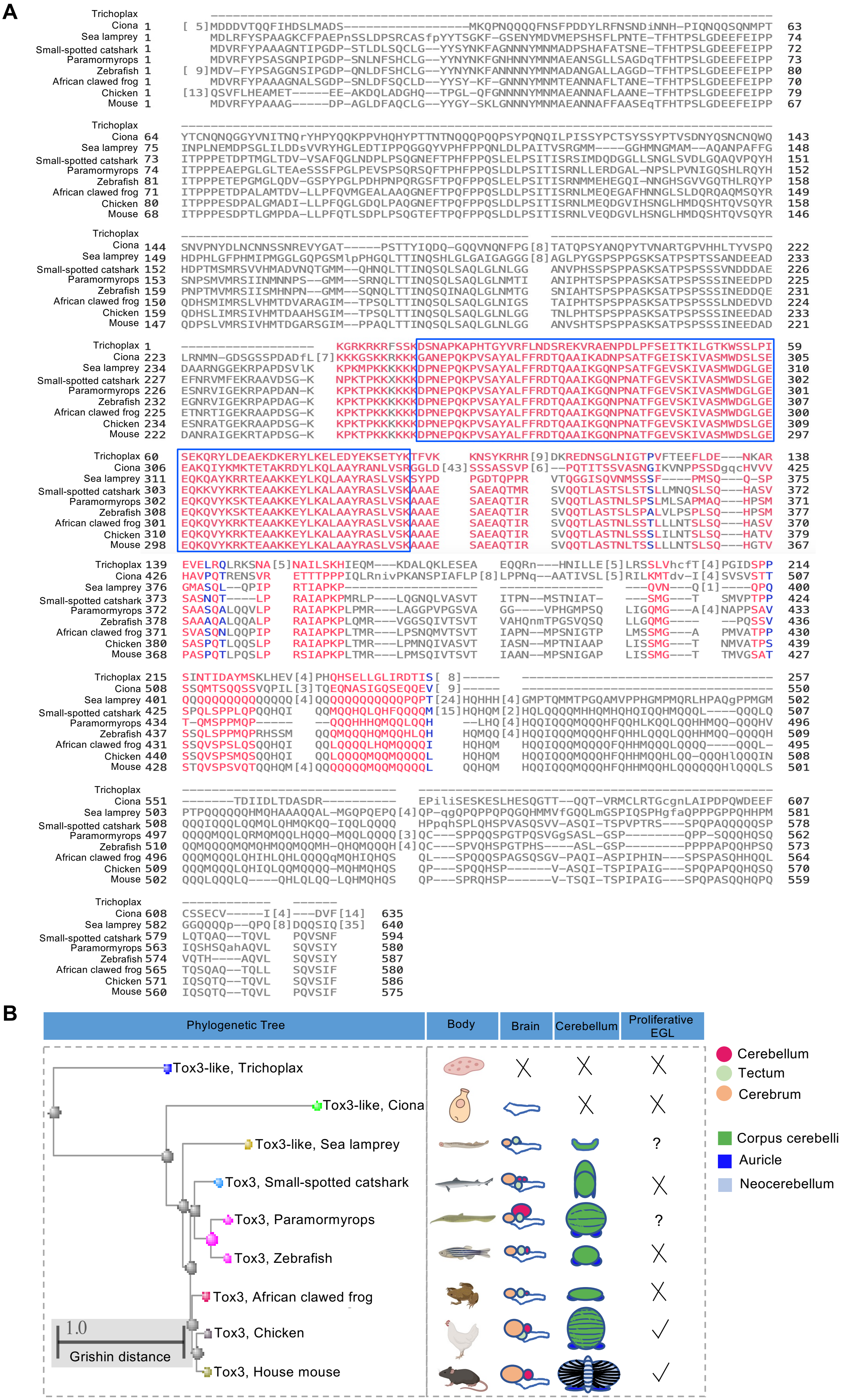
A
Trichoplax
Ciona
Sea lamprey
Small-spotted catshark
Paramormyrops
Zebrafish
African clawed frog
Chicken
Mouse
B
Phylogenetic Tree
Body
Brain
Cerebellum
Proliferative EGL
Tox3-like, Trichoplax
Tox3-like, Ciona
Tox3-like, Sea lamprey
Tox3, Small-spotted catshark
Tox3, Paramormyrops
Tox3, Zebrafish
Tox3, African clawed frog
Tox3, Chicken
Tox3, House mouse
1.0
Grishin distance
Cerebellum
Tectum
Cerebrum
Corpus cerebelli
Auricle
Neocerebellum

**Figure S7. Evolutionary analysis of Tox3 and Tox3-like proteins across representative vertebrate and basal metazoan species.**

(A) Multiple sequence alignment of Tox3 and Tox3-like proteins from nine representative species. The HMG-box domain is highlighted by purple boxes, and highly conserved residues are shown in red.

(B) Phylogenetic tree derived from the aligned sequences, illustrating clustering patterns and Grishin distances. Sequence alignment was performed using the NCBI Constraint-based Multiple Alignment Tool (COBALT), and the phylogenetic tree was generated using the Fast Minimum Evolution method. Protein accession numbers: Trichoplax (XP_002111752.1), Ciona (NP_001071666.2), Sea lamprey (XP_032813466.2), Small-spotted catshark (XP_038662637.1), Paramormyrops (XP_023701334.1), Zebrafish (XP_005169075.1), African clawed frog (XP_018113328.1), Chicken (XP_046755386.1), and House mouse (NP_766501.2).

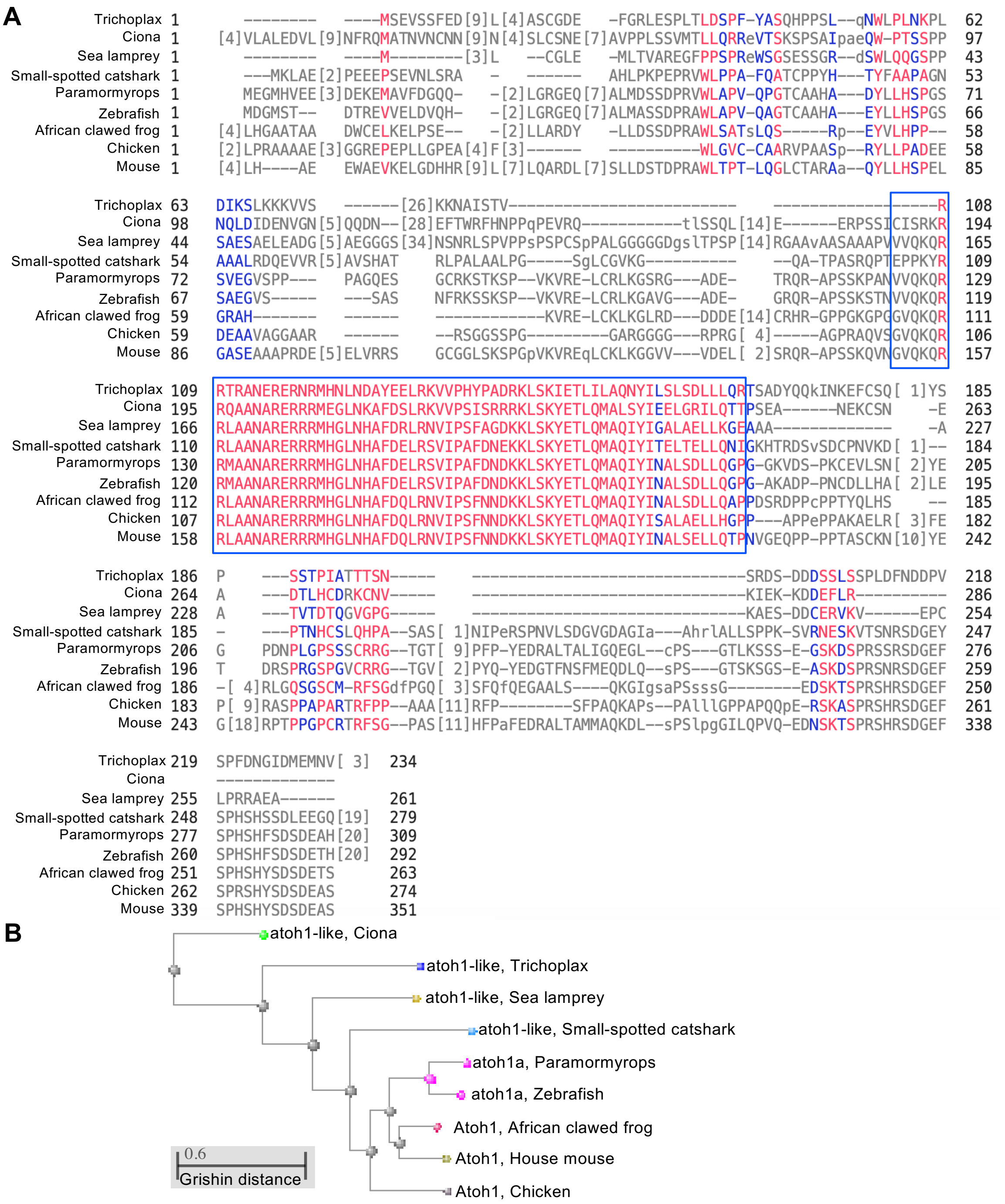


**Figure S8. Evolutionary analysis of Atoh1, atoh1a, and atoh1-like proteins across representative metazoan and vertebrate species.**

(A) Multiple sequence alignment of Atoh1, atoh1a, and atoh1-like proteins from nine representative species. The basic helix-loop-helix domain is highlighted by purple boxes, highly conserved residues are shown in red.

(B) Phylogenetic tree based on the aligned sequences, illustrating species clustering and Grishin distances. Sequence alignment was performed using the NCBI Constraint-based Multiple Alignment Tool (COBALT), and the phylogenetic tree was constructed using the Fast Minimum Evolution method. Protein accession numbers: Trichoplax (XP_002111626.1), Ciona (XP_002124707.3), Sean lamprey (XP_075921619.1), Small-spotted catshark (XP_038649396.1), Paramormyrops (XP_023692480.1), Danio rerio (NP_571166.2), African clawed frog (XP_018107642.1), Chicken (XP_004941187.3), and House mouse (NP_031526.1).

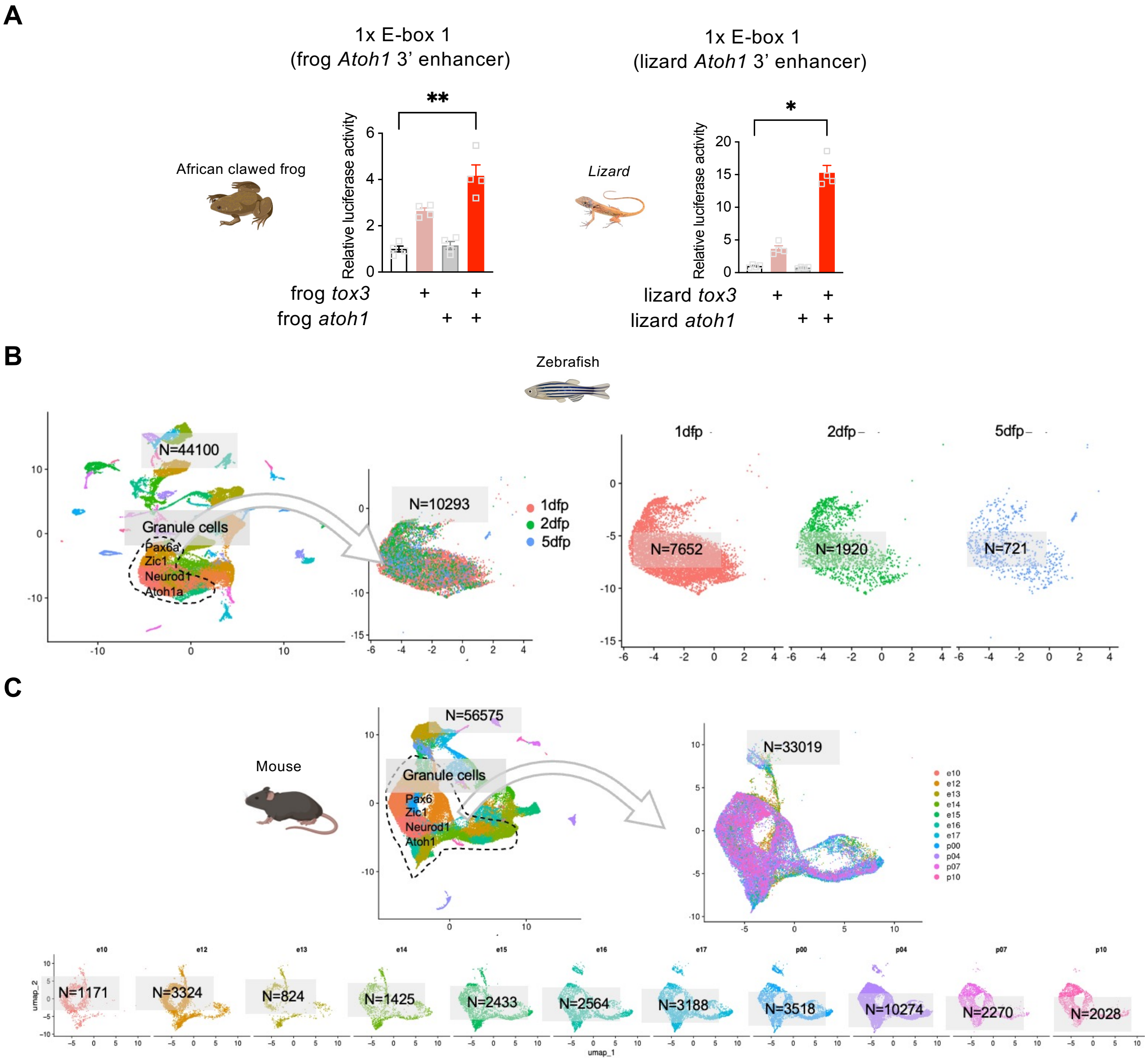


**Figure S9. Luciferase assays and single-cell transcriptomic analyses.**
(A) Luciferase assays showing that Tox3 and Atoh1 synergistically activate the conserved *Atoh1* 3' E-box1 in lizard. (B) Uniform Manifold Approximation and Projection (UMAP) of 44,100 cells from the developing zebrafish brain. Granule cells were identified by marker gene expression and subsetted from the full dataset. Granule cell clusters at each developmental are shown on the right. (C) UMAP embedding of 56,575 cells from the developing mouse cerebellum. Granule cells were identified based on marker gene expression and subsetted from the full dataset. Granule cell clusters at each developmental stage are shown below. Values are reported as mean ± SEM. *$P$<0.05, **$P$<0.01. Luciferase assay results are representative of three independent experiments.

## Supplementary Table

**Table S1:** Comparison of *Tox3* expression duration in the cerebellum across zebrafish, mouse and human.

| Species | *Tox3* Expression Period | Average Lifespan | *Tox3* expression (% Lifespan) |
|---|---|---|---|
| Zebrafish | ~5 days | ~3 years | ~0.45 |
| Mouse | ~4 weeks | ~2 years | ~3.84 |
| Human | ~2 years | ~70 years | ~2.86 |

## Movies

**Movie S1 (separate file):** Global deletion of *Tox3* results in severe ataxia, related to Figure 2. Video recordings of postnatal day 31 mice are shown. At the start of the video, the larger mouse in the center is *Tox3*$^{+/+}$, and the two smaller mice on either side are *Tox3*$^{-/-}$.

**Movie S2 (separate file):** Nervous system-specific deletion of *Tox3* leads to the onset of ataxia around postnatal day 10 (P10), related to Figure 2. Videos of P10 mice are shown. Left, *Tox3*$^{f/f}$; right, *Nestin-Cre;Tox3*$^{f/f}$.

**Movie S3 (separate file):** Nervous system-specific deletion of *Tox3* causes severe ataxia by postnatal day 14 (P14), related to Figure 2. Videos of P14 mice are shown. Left, *Tox3*$^{f/f}$; right, *Nestin-Cre;Tox3*$^{f/f}$.

## Datasets

**Dataset S1 (separate file)**: Z-scores showing the relative expression of transcription factors and cofactors across multiple organs. A total of 366, 272, 204, 208, 313, 190, and 353 genes were identified as enriched in the brain, cerebellum, heart, kidney, liver, ovary, and testis, respectively.

**Dataset S2 (separate file):** Secondary screening of cerebellum-enriched genes identified twenty-six candidate genes based on the following criteria: average Reads Per Kilobase per Million mapped reads (RPKM) > 1 and a postnatal day 0 (P0)/P63 expression ratio > 8.
